# Developing Best Practice Guidance for Discharge Planning Using the RAND/UCLA Appropriateness Method

**DOI:** 10.3389/fpsyt.2021.789418

**Published:** 2021-12-03

**Authors:** Natasha Tyler, Claire Planner, Matthew Byrne, Thomas Blakeman, Richard N. Keers, Oliver Wright, Paul Pascall Jones, Sally Giles, Chris Keyworth, Alexander Hodkinson, Christopher D. J. Taylor, Christopher J. Armitage, Stephen Campbell, Maria Panagioti

**Affiliations:** ^1^National Institute of Health Research (NIHR) Greater Manchester Patient Safety Translational Research Centre, Faculty of Biology, Medicine and Health, University of Manchester, Manchester, United Kingdom; ^2^National Institute for Health Research, School for Primary Care Research, School of Health Sciences, Faculty of Biology, Medicine and Health, University of Manchester, Manchester, United Kingdom; ^3^Division of Dentistry, Faculty of Biology, Medicine and Health, University of Manchester, Manchester, United Kingdom; ^4^Centre for Pharmacoepidemiology and Drug Safety, Division of Pharmacy and Optometry, School of Health Sciences, University of Manchester, Manchester, United Kingdom; ^5^Suicide, Risk and Safety Research Unit, Greater Manchester Mental Health National Health Service (NHS) Foundation Trust, Manchester, United Kingdom; ^6^Division of Population Health, Health Services Research and Primary Care, Centre for Primary Care and Health Services Research, The University of Manchester, Manchester, United Kingdom; ^7^School of Psychology, University of Leeds, Leeds, United Kingdom; ^8^Secondary Care Psychological Therapies Service, Pennine Care National Health Service (NHS) Foundation Trust, Bury, United Kingdom; ^9^Division of Psychology and Mental Health, Manchester Academic Health Science Centre, School of Health Sciences, Faculty of Biology, Medicine and Health, The University of Manchester, Manchester, United Kingdom; ^10^Manchester Centre for Health Psychology, University of Manchester, Manchester, United Kingdom; ^11^University National Health Service (NHS) Foundation Trust Manchester Academic Health Sciences Centre, Manchester, United Kingdom; ^12^National Institute of Health Research (NIHR) Manchester Biomedical Research Council, Manchester, United Kingdom

**Keywords:** mental health, RAND, discharge planning, care transitions, best practice, consensus methods, inpatient

## Abstract

**Background:** Discharge from acute mental health inpatient units is often a vulnerable period for patients. Multiple professionals and agencies are involved and processes and procedures are not standardized, often resulting in communication delays and co-ordination failures. Early and appropriate discharge planning and standardization of procedures could make inpatient care safer.

**Aim:** To inform the development of a multi-component best practice guidance for discharge planning (including the 6 component SAFER patient flow bundle) to support safer patient transition from mental health hospitals to the community.

**Methods:** Using the RAND/UCLA Appropriateness method, a panel of 10 professional stakeholders (psychiatrists, psychiatric nurses, clinical psychologists, pharmacists, academics, and policy makers) rated evidence-based statements. Six hundred and sixty-eight statements corresponding to 10 potential components of discharge planning best practice were rated on a 9-point integer scale for clarity, appropriateness and feasibility (median ≥ 7–9) using an online questionnaire then remote online face-to-face meetings.

**Results:** Five of the six “SAFER” patient flow bundle components were appropriate and feasible for inpatient mental health. One component, “Early Flow,” was rated inappropriate as mental health settings require more flexibility. Overall, 285 statements were rated as appropriate and feasible. Forty-four statements were considered appropriate but not feasible to implement.

**Discussion:** This consensus study has identified components of a best practice guidance/intervention for discharge planning for UK mental health settings. Although some components describe processes that already happen in everyday clinical interactions (i.e., review by a senior clinician), standardizing such processes could have important safety benefits alongside a tailored and timely approach to post-discharge care.

## Introduction

Safer discharge and transition from inpatient care to the community is a key global concern, with the World Health Organization Third Patient Safety Challenge featuring care transition as one of three priorities for action (1). Transition from acute mental health inpatient units to the community care is especially risky because multiple professionals and agencies are involved making communication delays and co-ordination failures likely to occur. Such communication and co-ordination failures lead to traumatic experiences for patients, and several adverse outcomes including patient safety incidents at pre- and post-discharge (2, 3). Early and appropriate discharge planning could make inpatient care safer and more person-centered, reduce unnecessary delays in hospital stays and contribute to a smoother adjustment of patients to the community after discharge (1, 4–7).

In the UK, one exemplar multicomponent discharge planning intervention developed by NHS Improvement is the SAFER Patient Flow Bundle (SAFER) (8) comprising of six key components: (1) Senior review (before midday); (2) Expected discharge date; (3) Clinical criteria for discharge; (4) Early assessments to improve patient flow; (5) Early discharge (aiming to discharge patients before midday); and (6) Multi-disciplinary review for patients with increased lengths of stay (8) (see Table 1, rows 1–6 for summary). Preliminary evidence obtained by case studies across the country shows that the SAFER patient flow bundle increases standardization of discharge planning procedures and results in reduced length of hospital stay, reduced discharge delays with no increase in complications, readmissions or contact with primary care, no reduction in patient satisfaction and an increase in staff satisfaction (8).

**Table 1:** Evidence sources informing potential intervention components (created by authors).

**Component**	**Summary of component**	**Source**
1. Clinical criteria for discharge	Clinical criteria for discharge has been used successfully to improve safety and patient flow at discharge in other clinical populations by providing biomarkers, or clinical criteria for patients to meet to be considered ready for discharge.	SAFER patient flow bundle
2. Estimated date of discharge	An estimated date of discharge (set at admission) has been used successfully to improve safety and patient flow at discharge in other clinical populations, as part of the SAFER bundle.	SAFER patient flow bundle
3. Early discharge	SAFER literature suggests that aiming for one third of all patients (due to be discharged on a particular day) to be discharged before midday is beneficial for patient flow, quality and safety.	SAFER patient flow bundle
4. Senior review	A review by a senior clinician each day has been used successfully to improve safety and patient flow at discharge in other clinical populations.	SAFER patient flow bundle
5. Early flow	Ensuring flow of patients will commence at the earliest opportunity from assessment units to inpatient wards and ensuring wards that routinely receive patients from assessment units will ensure the first patient arrives on the ward by 10 a.m., has been successful in improving patient flow, quality and safety in other clinical populations.	SAFER patient flow bundle
6. Multi-disciplinary team	Implementing MDTs for patients with extended length of stays (7 days in other clinical populations) has been successful in improving patient flow, quality and safety in other clinical populations according to the SAFER patient flow bundle literature.	SAFER patient flow bundle
7. Multi-agency team	The implementation of multi-disciplinary, multi-agency discharge teams within mental health trusts (including ward staff, community staff, emergency services, housing etc.) Our co-design workshop suggested multi-disciplinary, multi-agency discharge teams would improve continuity and reduce duplication between and within services.	(9)
8. Patient Written Discharge Plan	Our co-design workshop revealed that inter-agency multi-professional groups involved in mental health discharge processes, agreed that patient written discharge plans would improve safety, communication and continuity of care for patients discharged from acute mental health services.	(9, 10)
9. Improved Discharge Summary to Primary Care	The implementation of improved quality documentation sent to primary care when a patient is discharged from mental health inpatient settings. Improving the quality of discharge summaries has been suggested to effective in other clinical populations in improving safety and continuity of care.	Spencer (11, 12) and interviews
10. Social information Capture	Previous work that involved ethnography (observation) of professional processes around transitions of care in acute mental health, highlighted the importance of capturing certain categories of social information at discharge to reduce delayed discharge and improve safety.	Tyler admissions paper (under review BMC Psych)

SAFER is designed as a generic discharge planning intervention without targeting any specific setting/condition and therefore might fail to fully address the unique discharge planning challenges in mental health settings. Interviews with stakeholders (e.g., professionals, service users, families, and key informants) highlighted that SAFER needs to be significantly modified to in terms of content, timelines and staff roles for mental health settings (4, 13). Stakeholder also agreed some components could be excluded if not directly applicable or supplemented by additional useful components (4, 13). Improving inter-agency and multi-professional communication, information sharing and patient empowerment/shared decision making are key to improving safety in mental health care transitions (4, 9, 14, 15); however, these are not current components of the original SAFER patient flow bundle (4, 16). Therefore, consensus amongst experts is needed to understand how to operationalization the SAFER patient flow bundle for mental health and whether information-sharing components would further strengthen discharge planning best practice. Furthermore, as SAFER aligns with best practice guidelines, some inpatient mental health settings may already use some of its components (17), but the barriers that staff face implementing such best practice guidelines need to underpin any implementation plans.

The RAND/UCLA Appropriateness Method (RAM) is an internationally recognized consensus technique using a panel of experts to codify appropriate procedures or actions, presented as statements, relating to practice and policies (18). RAM methodology is typically applied to clinical practice, such as in the development of clinical guidelines and recommendations (19). More recently, a growing number of studies have illustrated that RAM can also be successfully used for developing policy and organizational/quality improvement interventions. For example RAM has been used to develop an intervention to support patients on sickness absence from work (20) and a psychological intervention delivered by telephone (21). RAM has also been used to identify “necessary” items for assessing and improving patient safety in general practice (19) and assess the appropriateness and feasibility of policies and strategies aimed at improving the retention of GPs (22).

The purpose of this study is to use the RAM (18) to identify components for a multi-component discharge planning intervention (based on existing best practice, i.e., SAFER patient flow bundle) to support safer patient transition from mental health hospitals to the community. RAM uses hundreds of individual statements; which will enable key stakeholders to decide exactly which components are appropriate and feasible and how they should be operationalized in mental health settings.

## Methods

We used the RAND/UCLA Appropriateness Method (RAM) (18) combining a systematic summary of available scientific evidence with the collective judgment of experts. This approach required panelists to rate the clarity, appropriateness and feasibility of statements relating to different components of a potential discharge planning intervention, using structured rating forms (18). The RAM aims to form a consensus opinion among experts, with individual opinions forming a refined, aggregated and group opinion. The study was approved by the UK Health Research Authority (HRA) and Health and Care Research Wales (20/NW/0228).

### Panelists

We recruited a case-mix sample of panelists who had expertise in mental health discharge, including psychiatrists, psychiatric nurses, clinical psychologists, pharmacists, academics and policy makers. The clinicians brought their knowledge and experiences working across primary and secondary care, including inpatient adult acute hospital settings and community mental health team settings. The mixed sample of panelists presented a wide variation of relevant views to mental health discharge.

In accordance with recommendations from the RAND Corporation, we aimed to recruit 9-10 panelists to allow for a focused discussion but with opportunity for different perspectives to be expressed. Following consultation with the research team, lead author NT contacted prospective panelists based in universities, inpatient mental health services, professional associations (i.e., Royal College of Psychiatrists) and third sector organizations/public bodies in England and Wales. Participants were identified due to their respective knowledge and informed consent was gathered before participation.

### Development of Statements (and Components)

Ten intervention components including 659 statements were developed from the evidence base and including the authors previous work: two systematic reviews (5); interviews with four stakeholder groups (patients *n* = 6, carers *n* = 7, mental health care professionals *n* = 14, and key informants *n* = 7); an NHS quality and safety improvement intervention [SAFER patient flow bundle (8)] and other discharge planning interventions that have demonstrated some degree of effectiveness, but have not been rigorously tested within mental health settings (8–11). The statements mapped on to 10 potential intervention components, see Table 1. The 10 potential intervention components consisted of the 6 components of the SAFER patient flow bundle: clinical criteria for discharge, estimated date of discharge, early discharge, senior review, early flow, multi-disciplinary team (8). The remaining 4 components were evidence-based interventions for improving information sharing in mental health care transitions (4, 9, 12, 16). There were 11 overarching statements representing the 10 components in Table 1, with “early flow” split into two (these were called K statements). The rationale behind each component was condensed into a 38-page evidence booklet that described the source, and key features of the included components (panelists were sent the evidence booklet to read prior to the commencement of round 1). Example K statements include: “Every patient is given Clinical Criteria for Discharge” and “A Patient Written Discharge Plan is developed for each patient.” Example detailed statements included “A nurse must capture every patients safeguarding status in the patient record at admission” and “Community carers must attend all discharge MDT meetings.”

### Consensus Procedures

This study was completed over two rounds. The first round comprised an online questionnaire and the second round three 2.5-h virtual meetings *via* Zoom. In both rounds, panelists were asked to rate each statement on a 9-point integer scale. Panelists were instructed to consider the “average” adult patient (18+), being discharged from the “average” inpatient mental health ward and under “average circumstances” in England and Wales when assigning their ratings.

In the first round, panelists were sent the online questionnaire *via* email in September 2020, and were asked to return the completed questionnaire within 2 weeks (the deadline was 1 week before the online meeting). Panelists were asked to rate the clarity of each statement and its appropriateness to facilitate safer discharge from an acute mental health care setting. Ratings for clarity ranged from 1, utterly unclear and ambiguous to 9, utterly clear and unambiguous. Ratings for appropriateness ranged from 1, unnecessary and always inappropriate (no exceptions) to 9, necessary and always appropriate (no exceptions). Panelists were invited to provide alternative wordings for the statements or suggest new statements.

An Excel spreadsheet was used to collate data from the first-round questionnaire. The frequency of each response on the 1–9 scale was collected for each statement and the median rating for each statement was calculated. The inter-percentile range adjusted for symmetry (IPRAS) technique was used to assess the level of agreement between the responses of the panelists for each statement and to construct a disagreement index (DI). The disagreement index is calculated by dividing the interpercentile range (IPR) by the interpercentile range adjusted for symmetry (IPRAS) (18). Any statement that had a DI > 1 showed disagreement within the panel. A DI < 1 demonstrated sufficient agreement between the panelists. These first round data collected were used to create personalized rating and for moderation of the sheets for the second, face-to-face round.

In the second round, panelists met once a week for 3 weeks (October 2020), under the chairmanship of two moderators (SC, Ahmed Hankir). SC had extensive previous experience of chairing RAM panels and Ahmed Hankir is a psychiatrist and academic with a large social media following in mental health. The two moderators had a sheet with each panelist's response for each of the statements, and the median score provided by the panel.

Each panelist received a personalized rating sheet that contained their own rating, and for comparison, presented the frequency distribution for the group ratings (anonymized) and the overall panel median rating from round one. Panelists used this information and discussed each statement as a group then independently re-rated the appropriateness of the statements and to rate the feasibility of statements on individual rating sheets. The “feasibility” scale asked panelists to rate how feasible it would be to implement the intervention in NHS settings in England and Wales, as this is the setting in which the intervention will initially be tested. Once again, panelists were able to propose alternative wordings for statements, which they could later refine by consensus decision.

Data from the second round were managed using an Excel spreadsheet and the median scores and DI calculated. Statements that had a median of 7 or greater and a disagreement index < 1 across the points of clarity, appropriateness and feasibility were included.

### Tabulating Results

We tabulated the final list of statements which mapped onto to the 10 potential intervention components and for each component outlined where there was consensus related to: (i) when the component should be delivered; (ii) what precisely is delivered; (iii) how the component is delivered; and (iv) who delivers it.

## Results

### Panelists

Of the 11 panelists who agreed to take part in the study, nine completed both rounds (the online questionnaire and the three virtual meetings). One panelist withdrew due to illness before receiving the online questionnaire. Another panelist was not able to attend the second virtual meeting due to an urgent clinical commitment and no ratings for this panelist were collected for statements in components 3–6.

The panel comprised of two psychiatrists, two mental health nurses, one pharmacist, one clinical psychologist, two key informant/policy makers and two academics (one service user researcher). The panelists had a range of inpatient and community experience. Five of the panelists who completed all rounds were men and four were women (see Supplementary Table 11 for details). It was estimated that each member of the panel committed 20 h of work to the consensus-building exercise.

### Statements Rated

A total of 668 statements were rated in the second round. This included nine rewordings, suggested by the panel. Of the 668 statements, 272 reflected six components of the SAFER patient flow bundle (criteria for discharge = 86; estimated date of discharge = 39; early discharge = 15; senior review = 27; early flow = 19; and multidisciplinary team meetings = 86) and 396 referred to the remaining 4 components (multi-agency teams = 41 patient written discharge plan = 87, primary care discharge summary = 170 and capture of patients' social information = 98), see Appendices 1, 2. Eleven overarching statements succinctly summarized all intervention components but without considering how these might be operationalized.

### Statements Rated as Appropriate and Feasible

One statement in the social information capture component *(Every patient's safeguarding status must be captured in the patient record at admission)* received the highest rating of appropriateness (median 9, DI < 1) and feasibility (median 9, DI < 1): see Appendix 1 and Table 2.

**Table 2:** Statements rated most highly—appropriate (median 9, DI < 1) and feasible (median ≥ 7, DI < 1) (created by authors).

**Component**	**SAFER patient flow bundle component**	**Statement number**	**Statement**
1. Clinical criteria for discharge	Yes	8	CCD must be developed individually for each patient (patient centered)
		14	CCD must be developed individually for each patient
		16	CCD must be developed around “goals” or “purpose” of admission
2. Estimated discharge date	Yes	79	EDD must be set for all patients
3. Early discharge	Yes	0	
4. Daily senior review	Yes	130	Weekly review about discharge must be conducted by approved clinician or nominated deputy in relation to discharge*
5. Early flow	Yes	0	
6. Multi-disciplinary discharge meeting	Yes	0	
7. Multi-agency team	No	0	
8. Patient written discharge plan	No	340	PWDP must include a contact phone number for help post-discharge (i.e., if I have problems I must call)
9. Primary care discharge summary	No	391	Patient name must be included on the PCDS
		392	Patient preferred name must be included on the PCDS
		393	Patient date of birth must be included on the PCDS
		395	Patient NHS number must be included on the PCDS
		416	Reason for admission must be included in the PCDS (i.e., he health problems and issues experienced by the patient that prompted the decision to admit to hospital)
		421	Discharge details must be included in the PCDS
		425	Date and time of discharge must be included in the PCDS
		445	Consent relating to child must be included in the PCDS (i.e., record of person with parental responsibility or appointed guardian where child lacks competency)
		450	Safeguarding issues must be included in the PCDS (i.e., any legal matters relating to safeguarding of a vulnerable child or adult, e.g., child protection plan, protection of vulnerable adult.)
		452	Risk to self must be included in the PCDS (i.e., any risk the patient poses to themselves- suicide, self-harm etc.)
		458	Person completing record must be included in the PCDS
		462	Date and time of completion of PCDS must be included
		477	Medication name must be included in the PCDS
		479	Medication quantity supplied on discharge must be included in the PCDS
		483	Dose directions description must be included in the PCDS (A single plain text phrase describing the entire medication dosage and administration directions, including dose quantity and medication frequency)
		484	Dose amount description must be included in the PCDS (A plain text description of medication single dose amount, e.g., 30 mg or 2 tabs)
		485	Dose timing description must be included in the PCDS (A plain text description of medication dose frequency e.g., Twice a day, at 8 a.m., 2 p.m., and 10 p.m.)
		486	Structured dose direction must be included in the PCDS (Recommendation of the time period for which the medication should be continued, including direction not to discontinue)
		512	Description of allergies or adverse reactions must be included in the PCDS
10. Social information capture		552	Every patient's accommodation status must be captured in the patient record at admission
		607	Every patient's physical healthcare needs must be captured in the patient record at admission
		626	Every patient's care giving responsibilities must be captured in the patient record at admission
		634	Details of patient's preferences about communication with informal carer must be captured at admission
		636	Every patient's involvement with other services (i.e., police, drug, and alcohol) must be captured in the patient record at admission
		648	Every patients General Practitioner details must be captured upon admission

Thirty-one statements were rated as appropriate (median 9, DI < 1) and feasible (median ≥ 7, DI < 1), of which 5 statements referred to three of the SAFER patient flow bundle components (criteria for discharge = 3; estimated discharge date = 1, senior review = 1) and 26 referred to additional components (patient written discharge plan = 1, primary care discharge summary = 19, and capture of patients' social information = 6), see Appendix 1 and Table 2.

A total of 253 statements were rated as appropriate (median ≥ 7, DI < 1) and feasible (median ≥ 7, DI < 1) of which 45 referred to four of the six SAFER patient flow bundle components (criteria for discharge = 19, estimated discharge date = 6, early discharge = 2, multi-disciplinary team meeting = 18) and 208 referred to additional components (multi-agency team = 11, patient written discharge plan = 49, primary care discharge summary = 116, social information capture = 32). None of the statements for the “early flow” component of the SAFER were rated as appropriate and feasible.

The complete list of 285 statements rated as appropriate (median ≥ 7, DI < 1) and feasible (median ≥ 7, DI < 1) is available, see Appendix 1.

### Statements Rated as Inappropriate and/or Infeasible

Forty-four statements were rated as appropriate (median ≥ 7, DI < 1) but with uncertain feasibility (median ≤ 6.5 ≥ 3.5, DI < 1) and 2 as appropriate (median ≥ 7, DI < 1) but infeasible (median ≤ 3, DI < 1). In total, 57 statements were rated as uncertain appropriateness (median ≤ 6.5, ≥ 3.5, DI < 1) but feasible, and 201 were rated as uncertain appropriateness (median ≤ 6.5, ≥ 3.5, DI < 1) and infeasible (median ≤ 3, DI < 1). Sixty-one statements were rated as inappropriate with uncertain feasibility and 18 statements were rated as inappropriate (median ≤ 3, DI < 1) and infeasible (median < 3, DI < 1). Seven statements had disagreement in the panel and were omitted, 1 on basis of clarity and 6 on basis of feasibility (DI > 1).

### Criteria for Including Components in the Intervention

The 285 statements rated as appropriate (median ≥ 7, DI < 1) and feasible (median ≥ 7, DI < 1) were tabulated, outlining where consensus was reached in terms of how the intervention components should be operationalized, see Appendix 3. We compared the results to the ratings for the 11 overarching K statements when selecting which components to include (See Table 1 for a summary of components). For example, if a K statement was rated as appropriate but with uncertain feasibility, the component was included if the panel had reached consensus in terms of how it could be operationalized. In total, 9 of the 10 components were included. Two were included but with significant modifications.

### The Included Components

**Figure 1:**
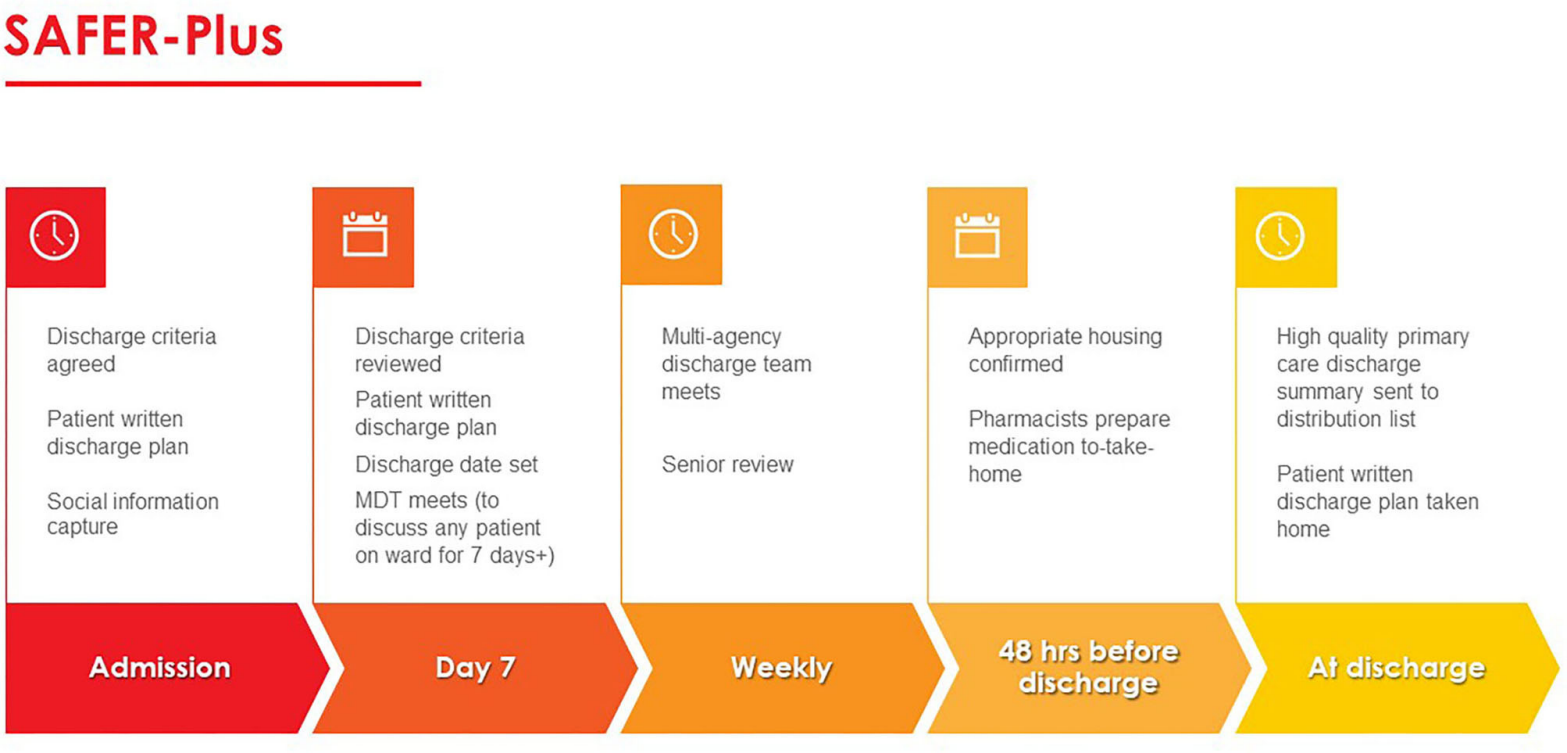
Core components of the intervention (created by authors).

Figure 1 outlines the patient pathway detailing which and when intervention components are delivered. The panel agreed that the component “*clinical criteria for discharge*” must be renamed as “*criteria for discharge*” and set for every patient, in line with the SAFER patient flow bundle. “Criteria for discharge” combine standardized and individualized items relating to social and generic criteria and focusing on “goals” or “purpose” of admission. The criteria are set at admission where possible. Also at admission *social and financial information* capture takes place with details added to the patient record and prompting action where required.

In line with the SAFER patient flow bundle, an *estimated discharge date* is agreed by a multidisciplinary discharge team in discussion with the patient. The *estimated discharge date* is communicated to patients when set and highlighted as a goal to work toward.

*Early discharge* is included, but with modifications. Early discharge is not required for one third of all patients (due to be discharged on a particular day) and a discharge before midday as per the SAFER patient flow bundle. Instead, ensuring housing and take-home medications are in place 48 h prior to the estimated date of discharge is used to facilitate an early discharge.

*Senior review* is also included with modification and instead of taking place daily, it was agreed that it should take place weekly, with a responsible clinician or nominated deputy.

A *Multi-disciplinary Discharge Team* review and *Multi-Agency Discharge Team* is established for every eligible patient with representatives from several community agencies.

Finally, a standardized and high quality Primary Care Discharge Summary (PCDS) which contains clear action points and a patient written discharge summary are produced for each discharged patient.

## Discussion

This study used RAM methodology with key stakeholder groups to identify which components of an existing multicomponent discharge planning interventions/guidance are applicable and feasible within mental health settings. This included how exactly each intervention component should be operationalized, to improve best practice and support safer discharge of patients from mental health hospitals to the community and how the intervention would work on a practical level with 285 statements rated as appropriate and feasible.

There was a general consensus among the panel that most of the proposed components are appropriate and feasible within mental health settings (9/10 components were included). The panel agreed that 5 of the 6 SAFER patient flow bundle components were appropriate and feasible for a mental health population, but that “Early Flow” (ensuring patients arrive on the ward as early as possible in the day) was not appropriate as mental health settings require more flexibility given that many admissions are unplanned, with patients sometimes admitted when experiencing a crisis which can be 24 h a day.

Many of the components within this bundle already happen in everyday clinical interactions (i.e., review by a senior clinician and social information capture). However, the panel agreed standardization of such processes could have important safety benefits. Furthermore, the panel agreed that this should not be at the detriment of patient-centeredness and this was particularly important for the “clinical criteria for discharge” and “estimated discharge date” components. This is in line with literature that suggested patient-centered approaches reduce readmissions and increase patient satisfaction (23, 24). Other components of the intervention are not typically standard practice generally or within mental health (i.e., patient written discharge plan and multi-agency team meetings) but the panel considered these useful additions to standard practice to improve care transition safety, and shared decision-making. The changes to the SAFER patient flow bundle outlined during this process enable higher quality practice in line with mental health mandates and guidelines such as recovery-oriented practice (25), for example by having a patient co-create a patient written discharge plan and shared-decision making mandates (26) by co-creating criteria for discharge with patients. The necessity to improve and standardize the quality of communication between clinicians and services (across health and social care) is a key safety concern for patients and families (4) as is improving the quality of communication between clinicians and service users during and after discharge (4).

Forty-four statements were considered appropriate for an intervention, but not feasible to implement, therefore commissioners and policy-makers need to consider the barriers to feasibility and how to make them operationable. Two of the nine components (early discharge and senior review) that the panel agreed should be included, needed significant modifications. As the output of this study is developed into an implementable intervention, it is important to consider what is feasible from a resource perspective. Using stakeholder engagement approaches and co-production will enable the intervention to be further adapted based on a focused discussion of potential implementation barriers. This is important as previous research using the RAM method for intervention development, has highlighted the potential disparity between “ideals” defined in such a method and reality in the context of providing individualized care (27).

This work focuses on discharge planning that happens only within an inpatient setting, however there is a large body of literature comprising of interventions that improve the quality of safety of care transitions beyond the inpatient setting, for example there has been considerable work by Forchuk et al. concerning therapeutic relationships that continue from hospital to community (28, 29). To avoid further fragmentation of care and better joined-up care, it is important to understand how discharge planning interventions/practice align with community follow-up interventions/practice.

### Research and Policy Implications of Using RAM for Developing Best Practice Interventions/Guidance

This work, a RAM method, whereby professionals and researchers to provide their expert opinions about best practice, sits within a wider project. The RAM method provides a systematic approach to developing face valid components of a discharge planning intervention in acute mental health settings but further testing is required to fully understand acceptability, reliability, validity, and implementation issues (30). The RAM method is not an end, it is a means to an end, to develop a quality/safety intervention in combination with other methods e.g., co-production with patients/carers to further refine this intervention. Patients and carers/relatives have been involved throughout the wider project from the planning stages, including statement development and we will continue to work with them as we refine the intervention based on the RAM findings. Similarly to others who have used the RAM approach for intervention development (20, 31), we found it to be a systematic method for assessing the initial acceptability of the proposed intervention components. The systematic assessment of evidence-based statements delivers clarity in terms of what professionals agree is appropriate and feasible in the complex context of mental health services. This was demonstrated in the results whereby some intervention components were rated appropriate with uncertain feasibility (i.e., multi-agency team meetings); in these cases the individual statements highlighted areas of consensus concerning intricacies of delivery. RAM was also particularly useful in providing consensus on the preferred content and structure of individual elements of practice. For example, for the estimated discharge date component of the intervention (EDD), there was consensus that an EDD should be co-decided by a multi-disciplinary team which includes the patient (and the carer after gaining patient consent), and should be described to the patient as a goal to work toward (albeit amenable to change rather than rigidly set). Moreover, RAM was also a practical and convenient approach to use during the Covid-19 pandemic where face-to-face interaction is limited, enabling busy clinicians and professionals to contribute at a time and location that suited them (for the online discussions). Round 1 is always conducted remotely, irrespective of social distancing requirements but we chose to conduct round 2 using video technology. This modification enabled people who might otherwise have been unable to attend due to travel and commitments to contribute from any location, reducing also the cost of traditional RAM.

### Strengths and Limitations

This study presents a comprehensive method of intervention development, whereby a multi-professional panel of expert stakeholders rigorously rated, discussed and re-rated over 600 statements relating to 10 potential components of a discharge planning intervention in acute mental health settings. The agreed components of the intervention reflected the perspectives of all key stakeholder professional groups relevant to the settings and intervention. The included statements were informed by a systematic summary of available scientific evidence combined with the collective judgment of experts. The RAM consensus method aimed to provide face validity; which is an excellent starting point for further development.

However, this study also has important limitations. Due to the precise, clinical focus of the statements, requiring an expert knowledge of health system procedures and processes, panelists were professionals and researchers with expertise in mental health discharge. However, recognizing the value of the service user and carer perspective, we have continually involved these groups in the wider project, including statement development for the RAM, the next stage in this process is to refine the intervention based on the RAM outcomes in planned co-production workshops with patients and carers. We included one panelist who had lived experience however his primary role was as a service-user researcher) and also one of the chairs contributed a lived experience alongside his clinical “lens” in facilitating the discussion.

Mental health care transitions, by nature are complex, individualized and often involve the co-ordination of multiple services. RAM panels typically include 9 individuals based in the UK, therefore the opinions of a single panel may not be representative of all clinicians, researchers and policy-makers involved across the complex care pathway in the UK and particularly in mental health settings outside the UK. However, panel sizes of 9 to 12 members provide results that are typically reproduced by a second panel (32). The completely remote RAM also had some limitations, panelists had to attend three meetings instead of one, and one panelist could not make them all. It is also unclear how or whether the quality of discussion was as good as it would have been face-to-face.

### Future Direction

We will conduct further engagement with a wider range of stakeholders, particularly patients, informed by appropriate methodologies for stakeholder engagement and co-production to refine interventions produced using RAM before an empirical evaluation (i.e., feasibility randomized controlled trial).

## Conclusion

Mental health care transitions are a critical, vulnerable stage in a complex care pathway with serious safety threats and potential adverse outcomes for patients (such as suicide and self-harm). The use of RAM has enabled us to develop a preliminary but clearly outlined model of a multicomponent discharge planning intervention focusing specifically on what multi-professional stakeholders agree is not only appropriate but also feasible to implement in the UK mental health settings. The application of RAM therefore has provided an evidence-based guidance to facilitate the development of a discharge planning intervention, which can be implemented as standard/best practice to enable sustainable improvement. The next important step is that patient voices are captured in the development of such best practice interventions.

## Data Availability Statement

The original contributions presented in the study are included in the article/Supplementary Material, further inquiries can be directed to the corresponding author.

## Ethics Statement

The studies involving human participants were reviewed and approved by HRA and Health and Care Research Wales. The patients/participants provided their written informed consent to participate in this study. Written informed consent was obtained from the individual(s) for the publication of any potentially identifiable images or data included in this article. All panelists provided written consent prior to their participation in the study.

## Author Contributions

MP, NT, and SC conceived the study. MP provided overall guidance and management of the study. SC chaired the panel and provided expert guidance in regards to the methodology. TB and SG also provided methodological, clinical, and patient safety guidance. NT, PP, and OW developed the statements and the panel guidance document and NT devised the panel. MB and AH conducted the statistical analysis. CP also analyzed the results of the study. CA and CK provided oversight in regards to the behavioral science, to enable this to be included in intervention development going forward. RK helped with recruitment and provided expertise from a pharmacological perspective. CT provided expertise from a clinical perspective. CP, NT, and MP devised the manuscript, with contributions from all authors in their relevant fields of expertise. All authors contributed to the article and approved the submitted version.

## Funding

This work was funded by the National Institute for Health Research (NIHR) Greater Manchester Patient Safety Translational Research Centre (award number: PSTRC-2016-003).

## Author Disclaimer

The views expressed are those of the author(s) and not necessarily those of the NIHR or the Department of Health and Social Care (Award Number: PSTRC-2016-003).

## Conflict of Interest

The authors declare that the research was conducted in the absence of any commercial or financial relationships that could be construed as a potential conflict of interest.

## Publisher's Note

All claims expressed in this article are solely those of the authors and do not necessarily represent those of their affiliated organizations, or those of the publisher, the editors and the reviewers. Any product that may be evaluated in this article, or claim that may be made by its manufacturer, is not guaranteed or endorsed by the publisher.

## References

[1] World Health Organisation (WHO). WHO | The third WHO Global Patient Safety Challenge: Medication Without Harm. WHO World Health Organization (2019). Available online at: https://www.who.int/patientsafety/medication-safety/en/ (accessed March 19, 2020).

[2] Wright N Rowley E Chopra A Gregoriou K Waring J. From admission to discharge in mental health services: a qualitative analysis of service user involvement. Heal Expect. (2016) 19:367–76. 10.1111/hex.12361 25817297 PMC5055255

[3] McGowan I. National confidential inquiry into suicide and homicide by people with mental illness. In: A Companion to Criminal Justice, Mental Health & Risk. (2014). Available online at: www.hqip.org.www.bbmh.manchester.ac.uk/cmhs (accessed August 8, 2018).

[4] Tyler N Wright N Panagioti M Grundy A Waring J. What does safety in mental health care transitions mean for service users and other stakeholder groups: An open-ended questionnaire study. Heal Expect. (2021) 24(Suppl. 1):185–94. 10.1111/hex.13190 33471958 PMC8137494

[5] Tyler N Wright N Waring J. Interventions to improve discharge from acute adult mental health inpatient care to the community: systematic review and narrative synthesis. BMC Health Serv Res. (2019) 19:883. 10.1186/s12913-019-4658-0 31760955 PMC6876082

[6] Steffen S Kösters M Becker T Puschner B. Discharge planning in mental health care: a systematic review of the recent literature. Acta Psychiatr Scand. (2009) 120:1–9. 10.1111/j.1600-0447.2009.01373.x 19486329

[7] NICE. Transition Between Inpatient Hospital Settings and Community or Care Home Settings for Adults with Social Care Needs | Guidance and Guidelines | NICE. National Institute for Health and Care Excellence (2015).

[8] NHS Improvement. Rapid Improvement Guide: the SAFER Patient Flow Bundle. (2016). Available online at: https://improvement.nhs.uk/resources/rapid-improvement-guide-safer-patient-flow-bundle/ (accessed November 12, 2019).

[9] Tyler N Wright N Grundy A Gregoriou K Campbell S Waring J. Co-designing a mental health discharge and transitions of care intervention: a modified nominal group technique. Front Psychiatry. (2020) 11:328. 10.3389/fpsyt.2020.00328 32372990 PMC7186904

[10] Jack B Paasche-Orlow M Mitchell S Forsythe S Martin J Brach C. Re-Engineered Discharge (RED) Toolkit. AHRQ Publ No 12. Rockville, MA (2013).

[11] eDischarge, Summary 2,.1 - PRSB. Available online at: https://theprsb.org/standards/edischargesummary/ (accessed July 29, 2020).

[12] Spencer RA Spencer SEF Rodgers S Campbel SM Avery AJ. Processing of discharge summaries in general practice: a retrospective record review. Br J Gen Pract. (2018) 68:e576–85. 10.3399/bjgp18X697877 29914879 PMC6058631

[13] Tyler N Daker-White G Grundy A Quinlivan L Armitage C Campbell S. Effects of the first COVID-19 lockdown on quality and safety in mental healthcare transitions in England. BJPsych Open. (2021) 7:e156. 10.1192/bjo.2021.996 34493959 PMC8410739

[14] Rowley E Wright N Waring J Gregoriou K Chopra A. Protocol for an exploration of knowledge sharing for improved discharge from a mental health ward. BMJ Open. (2014) 4:e005176–e005176. 10.1136/bmjopen-2014-005176 25273812 PMC4185338

[15] Slade M. Implementing shared decision making in routine mental health care. World Psychiatry. (2017) 16:146–53. 10.1002/wps.20412 28498575 PMC5428178

[16] Tyler N Wright N Gregoriou K Waring J. Improving mental health care transitions through information capture during admission to inpatient mental health services: a quality improvement study. BMC Health Serv Res. (2021) 21:1132. 10.1186/s12913-021-07136-2 34674690 PMC8529804

[17] National Institute for Health Care Excellence. Transition Between Inpatient Mental Health Settings and Community or Care Home Settings | Guidance and Guidelines | NICE. NICE (2016). Available online at: https://www.nice.org.uk/guidance/ng53 (accessed December 20, 2018).

[18] Fitch K Bernstein S Aguilar M Burnand B. The RAND/UCLA Appropriateness Method User's Manual. (2001). Available online at: https://apps.dtic.mil/docs/citations/ADA393235 (accessed November 12, 2019).

[19] Bell BG Spencer R Avery AJ Campbell SM. Tools for measuring patient safety in primary care settings using the RAND/UCLA appropriateness method. BMC Fam Pract. (2014) 15:110. 10.1186/1471-2296-15-110 24902490 PMC4060097

[20] Wright C Moseley A Chilvers R Stabb L Campbell JL Richards SH. Development of an early intervention to prevent long-term incapacity for work: using an online RAND/UCLA appropriateness method to obtain the views of general practitioners. Prim Health Care Res Dev. (2009) 10:65. 10.1017/S146342360800094730886898

[21] Faija CL Gellatly J Barkham M Lovell K Rushton K Welsh C. Enhancing the behaviour change wheel with synthesis, stakeholder involvement and decision-making: a case example using the ‘Enhancing the quality of psychological interventions delivered by telephone' (EQUITy) research programme. Implement Sci. (2021) 161:1–11. 10.1186/s13012-021-01122-2 33990207 PMC8120925

[22] Chilvers R Richards SH Fletcher E Aylward A Dean S Salisbury C. Identifying policies and strategies for general practitioner retention in direct patient care in the United Kingdom: a RAND/UCLA appropriateness method panel study. BMC Fam Pract. (2019) 201:1–12. 10.1186/s12875-019-1020-x 31514728 PMC6743144

[23] Thornicroft G Bebbington P Leff J. Outcomes for long-term patients one year after discharge from a psychiatric hospital. Psychiatr Serv. (2005) 56:1416–22. 10.1176/appi.ps.56.11.1416 16282261

[24] (UK) NCC for MH. Interventions to Improve Service User Experience. (2012). Available online at: https://www.ncbi.nlm.nih.gov/books/NBK327299/ (accessed July 2, 2021).

[25] Care Quality Commission. Brief Guide: Recovery Orientated Practice. (2020). Available online at: https://www.nice.org.uk/guidance/qs14 (accessed November 4, 2021).

[26] Health Social Care Act 2012.. (2015). Available online at: https://www.google.com/search?q=health+and+social+care+act+2012&rlz=1C1GCEA_enGB873GB873&oq=Health+and+Social+Care+Act+2012&aqs=chrome.0.0i512l10.993j0j7&sourceid=chrome&ie=UTF-8 (accessed November 4, 2021).

[27] Prys-Picard CO Campbell SM Ayres JG Miles JF Niven RM. Defining and investigating difficult asthma: developing quality indicators. Respir Med. (2006) 100:1254–61. 10.1016/j.rmed.2005.10.013 16303294

[28] Forchuk C Martin ML Chan YL Jensen E. Therapeutic relationships: From psychiatric hospital to community. J Psychiatr Ment Health Nurs. (2005) 12:556–64. 10.1111/j.1365-2850.2005.00873.x 16164506

[29] Forchuk C Reynolds W Sharkey S Mary-Lou M Jensen E. Transitional discharge based on therapeutic relationships: state of the art. Arch Psychiatr Nurs. (2007) 21:80–6. 10.1016/j.apnu.2006.11.002 17397689

[30] Campbell SM. Braspenning J, Hutchinson A, Marshall MN. Research methods used in developing and applying quality indicators in primary care. Qual Saf Health Care. (2002) 11:358–64. 10.1136/qhc.11.4.358 12468698 PMC1758017

[31] Hirsch JA Beall DP Chambers MR Andreshak TG Brook AL Bruel BM. Management of vertebral fragility fractures: a clinical care pathway developed by a multispecialty panel using the RAND/UCLA Appropriateness Method. Spine J. (2018) 18:2152–61. 10.1016/j.spinee.2018.07.025 30096377

[32] Shekelle PG Kahan JP Bernstein SJ Leape LL Kamberg CJ Park RE. The reproducibility of a method to identify the overuse and underuse of medical procedures. N Engl J Med. (1998) 338:1888–95. 10.1056/NEJM199806253382607 9637810

