# Supplementary material for: Developing Best Practice Guidance for Discharge Planning Using the RAND/UCLA Appropriateness Method
Source: Front Psychiatry. 2021 Dec 3;12:789418. doi: 10.3389/fpsyt.2021.789418 (PMC8680088; doi:10.3389/fpsyt.2021.789418)
Supplement: Supplementary file 1 [file Data_Sheet_1.docx]

**Supplementary file**

**Table 1. Criteria for discharge: statements grouped by ranking**

| **Ranking score** | **Statement number** | **Statement** |
| --- | --- | --- |
| Appropriate (median 9, DI <1) and feasible (median 9, DI <1) |  |  |
|  | N/A | No statements ranked in this category |
|  |  |  |
| Appropriate (median 9, DI <1) and feasible (median ≥7, DI <1) |  |  |
|  | 8 | CCD must be developed individually for each patient (patient centred) |
|  | 14 | CCD must be developed individually for each patient |
|  | 16 | CCD must be developed around 'goals' or 'purpose' of admission |
| Appropriate (median ≥7, DI <1) and feasible (median ≥7, DI <1) |  |  |
|  | 2 | CCD must be set upon admission for patients who are known to services |
|  | 10 | Patients must be involved in deciding upon Criteria for Discharge |
|  | 11 | CCD must be renamed 'Criteria for Discharge' to include relevant social and non-clinical factors |
|  | 15 | CCD must be a combination of standard items and individualised items |
|  | 18 | CCD must discuss locality of patient who is being discharged (higher rate of suicide for out of area patients) |
|  | 19 | CCD must include suicide risk and scoring |
|  | 20 | CCD must not include specific targets, but rather reflect mutual decisions from patient and practitioner |
|  | 21 | CCD must be set by an MDT team |
|  | 45 | CCD must be reviewed 7 days after being put in place |
|  | 56 | Having safeguarding issues addressed must be a CCD |
|  | 64 | Patient being mentally stable/no longer in acute crisis according clinician's judgement (i.e. self-harm) must be a CCD |
|  | 76 | Personal recovery must be part of CCD |
|  | 4 | CCD must be set by the nominated nurse in charge within the first week after admission |
|  | 9a | CCD must be set according to reason for admission |
|  | 51 | Carers must be involved in the development of CCD following patient consent |
|  | 52 | Carers must be informed of CCD when they set following patient consent |
|  | 57 | Having financial responsibilities/difficulties discussed (that may affect discharge) must be a CCD |
|  | 61 | Having physical healthcare needs reviewed must be a CCD |
|  | k1 | K1.  Every patient is given Clinical Criteria for Discharge (CCD) |
| Appropriate (median ≥7, DI <1) but with uncertain feasibility (median ≤6.5 ≥3.5, DI <1) |  |  |
|  | 1 | CCD must be set upon admission for all patients |
|  | 3 | CCD must be set by a consultant |
|  | 55 | Having appropriate accommodation to be discharged to must be a CCD |
|  | 59 | Informing involved services/agencies about a patients discharge must be a CCD |
|  | 60 | Having caring responsibilities acknowledged and addressed must be a CCD |
|  | 62 | Resolving unmet community needs must be a CCD |
|  | 65 | Patient being mentally stable/settled and no longer in acute crisis based on scores from standard tools must be a CCD |
|  | 73 | Patient having improved overall mental health functioning according to clinician's judgement must be a CCD |
| Appropriate (median ≥7, DI <1) but infeasible (median ≤3, DI <1) |  |  |
|  | 57 | Having financial responsibilities/difficulties addressed (that may affect discharge) must be a CCD |
|  | 61 | Having physical healthcare needs met must be a CCD |
| Uncertain appropriateness (median ≤6.5, ≥3.5, DI <1) but feasible |  |  |
|  | 9 | CCD must be set according to diagnosis/reason for admission |
|  | 17 | CCD must be set after diagnosis for patients unknown to services |
|  | 22 | CCD must be set in the patient's first MDT meeting |
|  | 23 | CCD must be set by the last MDT meeting (before expected discharge date) |
|  | 24 | CCD must be set in the first meeting with consultant |
|  | 33 | CCD must be set within 7 days of admission onto the ward for patients who are known to services |
|  | 34 | CCD must be set within 7 days of admission onto the ward for all involuntary admissions |
|  | 35 | CCD must be set within 7 days of admission onto the ward for all patients |
|  | 36 | CCD must be set within 7 days of admission onto the ward for all voluntary patients |
|  | 46 | CCD must be reviewed 1 month after being put in place |
|  | 75 | Patient having improved overall cognitive functioning according to clinician's judgement must be a CCD |
|  | 9b | CCD must be set according to diagnosis upon admission |
| Uncertain appropriateness (median ≤6.5, ≥3.5, DI <1) with uncertain feasibility (median ≤6.5, ≥3.5, DI <1) |  |  |
|  | 4 | CCD must be set by senior nurses |
|  | 7 | CCD must be set by junior doctors (Speciality training ST4 up to consultant) |
|  | 13 | CCD must be presented to family/carer for discussion of social circumstances |
|  | 25 | CCD must be set within 24 hours of admission to the ward for patients who are known to services |
|  | 26 | CCD must be set within 24 hours of admission to the ward for all involuntary admissions |
|  | 27 | CCD must be set within 24 hours of admission to the ward the for all patients |
|  | 28 | CCD must be set within 24 hours of admission to the voluntary patients |
|  | 29 | CCD must be set within 48 hours of admission to the ward for patients who are known to services |
|  | 30 | CCD must be set within 48 hours of admission to the ward for all involuntary admissions |
|  | 31 | CCD must be set within 48 hours of admission to the ward for all patients |
|  | 32 | CCD must be set within 48 hours of admission to the ward for all voluntary patients |
|  | 48 | CCD for phased leave must be set at admission |
|  | 49 | CCD for phased leave must be set within 7 days of admission |
|  | 50 | CCD for phased leave must be set within 48 hours of admission |
|  | 54 | Having (social/professional) networks in place must be a CCD |
|  | 58 | Ensuring patients benefits (financial) are correct must be a CCD |
|  | 63 | Stabilised medication use as determined following a medication review conducted by a pharmacist must be a CCD |
|  | 67 | Patient having the ability to recognise one's own mental illness (insight) based on scores from standard psychometric measurement tools must be a CCD |
|  | 71 | Patient having improved mental health symptoms according to clinician's judgement must be a CCD |
|  | 72 | Patient having improved mental health symptoms based on scores from standardised tools (e.g. depression scale) must be a CCD |
|  | 74 | Patient having improved overall mental health functioning based on scores from standardised tools must be a CCD |
|  | 77 | Using 'CHIME' as a framework to assess personal recovery must be considered when developing CCD |
|  | 78 | Psychometric measures that are found to correlate with relevant elements of the CHIME recovery conceptual framework must be used as CCD |
| Inappropriate (median ≤3, DI <1) and infeasible (median <3, DI <1). |  |  |
|  | 53 | Having carers available must be a CCD |

**Table 2. Estimated date for discharge: statements grouped by ranking**

| **Ranking score** | **Statement number** | **Statement** |
| --- | --- | --- |
| Appropriate (median 9, DI <1) and feasible (median 9, DI <1) |  |  |
|  | N/A | No statements ranked in this category |
| Appropriate (median 9, DI <1) and feasible (median ≥7, DI <1) |  |  |
|  | 79 | EDD must be set for all patients |
| Appropriate (median ≥7, DI <1) and feasible (median ≥7, DI <1) |  |  |
|  | 85 | EDD must be communicated to carers after gaining patient consent |
|  | 87 | EDD must be communicated to patients as soon as its set |
|  | 103 | EDD must be set by a multi-disciplinary team (MDT) |
|  | 108 | All patients should be involved in discussions when setting EDD |
|  | 109 | EDD must be formally highlighted to the patient as a goal to work towards that is always amenable to changes |
|  | k2 | K2. Every patient is given an Estimated Discharge Date (EDD) |
| Appropriate (median ≥7, DI <1) but with uncertain feasibility (median ≤6.5 ≥3.5, DI <1) |  |  |
|  | 92 | EDD must be set within 48 hours of admission |
|  | 93 | EDD must be set within 72 hours of admission |
|  | 104 | EDD must be set during the patient's first MDT meeting |
| Appropriate (median ≥7, DI <1) but infeasible (median ≤3, DI <1) |  |  |
|  | N/A | No statements ranked in this category |
| Uncertain appropriateness (median ≤6.5, ≥3.5, DI <1) but feasible |  |  |
|  | 82 | EDD must be set for voluntary patients |
|  | 83 | EDD must be set for involuntary patients |
|  | 94 | EDD must be set within 7 days of admission |
|  | 95 | EDD must be set within 14 days of admission |
|  | 96 | EDD must be set within 21 days of admission |
| Uncertain appropriateness (median ≤6.5, ≥3.5, DI <1) with uncertain feasibility (median ≤6.5, ≥3.5, DI <1) |  |  |
|  | 84 | EDD must be communicated to carers |
|  | 97 | EDD must be set 14 days before discharge (if patient has a longer stay than this) |
|  | 98 | EDD must be set 7 days before discharge (if patient has a longer stay than this) |
|  | 99 | EDD must be set 72 hours before discharge |
|  | 100 | EDD must be set 48 hours before discharge |
|  | 101 | EDD must be set 48 hours before discharge |
|  | 105 | EDD must be set by the patient's responsible clinician (if appropriate) or a senior clinician |
|  | 110 | EDD can be set by a senior nurse supervised by a senior psychiatrist |
|  | 111 | EDD can be set by a junior doctor supervised by a senior psychiatrist |
|  | 112 | If the EDD is set by anybody other than a senior psychiatrist, they must have received formal training |
|  | 113 | If the EDD is set by anybody other than a senior psychiatrist, they must be supervised *HOW MUCH* |
|  | 115 | EDD can be set by a junior doctor (or Speciality training ST1-3) |
|  | 116 | EDD can be set by a junior doctor (Speciality training ST4 up to consultant) |
| Inappropriate (median ≤3, DI <1) and infeasible (median <3, DI <1). |  |  |
|  | 81 | EDD must be set for all patients except older adults |
|  | 91 | EDD must be set within 24 hours of admission |
|  | 107 | EDD must be set at clerking meeting if patient is unknown to services |

**Table 3. Early discharge: statements grouped by ranking**

| **Ranking score** | **Statement number** | **Statement** |
| --- | --- | --- |
| Appropriate (median 9, DI <1) and feasible (median 9, DI <1) |  |  |
|  | N/A | No statements ranked in this category |
| Appropriate (median 9, DI <1) and feasible (median ≥7, DI <1) |  |  |
|  | N/A | No statements ranked in this category |
| Appropriate (median ≥7, DI <1) and feasible (median ≥7, DI <1) |  |  |
|  | 125 | To enable ED, pharmacists should be informed 48 hours before expected discharge date, so that medication TTOs should be prepared |
|  | 122 | To enable ED, confirm appropriate housing in place at least 48 hours before expected discharge date |
| Appropriate (median ≥7, DI <1) but with uncertain feasibility (median ≤6.5 ≥3.5, DI <1) |  |  |
|  | 122 | To enable ED, housing needs should be reassessed at least 48 hours before expected discharge date |
|  | 123 | To ED, housing needs should be reassessed at least 72 hours before expected discharge date |
|  | k3 | K3. Early discharge (ED) for one third of all patients (due to be discharged on a particular day); must be discharged before midday |
| Appropriate (median ≥7, DI <1) but infeasible (median ≤3, DI <1) |  |  |
|  | N/A | No statements ranked in this category |
| Uncertain appropriateness (median ≤6.5, ≥3.5, DI <1) but feasible |  |  |
|  | N/A | No statements ranked in this category |
| Uncertain appropriateness (median ≤6.5, ≥3.5, DI <1) with uncertain feasibility (median ≤6.5, ≥3.5, DI <1) |  |  |
|  | 117 | Targets must be set to achieve ED for 33% of patients (due to be discharged that day) |
|  | 118 | Ward staff should be aware that it is good practice to conduct ED for at least 33% of patients, but this should not be an audited target |
|  | 119 | Targets must be set to ED for 20% of patients (due to be discharged that day) |
|  | 120 | Ward staff should be aware that it is good practice to conduct ED for at least 20% of patients but this should not be an audited target |
|  | 121 | To enable ED, housing needs should be reassessed at least 24 hours before expected discharge date |
|  | 124 | To enable ED, pharmacists should be informed 24 hours before expected discharge date, so that medication TTOs should be prepared |
|  | 126 | To enable ED, pharmacists should be informed 24 hours before expected discharge date, so that medication TTOs should be prepared |
|  | 127 | Audits should be conducted and data recorded in regards to the numbers of ED conducted |
|  | 128 | Patients with multiple needs (i.e. service engagement) within the community should be prioritised for ED |
| Inappropriate (median ≤3, DI <1) and infeasible (median <3, DI <1). |  |  |
|  | N/A | No statements ranked in this category |

**Table 4. Daily senior review: statements grouped by ranking**

| **Ranking score** | **Statement number** | **Statement** |
| --- | --- | --- |
| Appropriate (median 9, DI <1) and feasible (median 9, DI <1) |  |  |
|  | N/A | No statements ranked in this category |
| Appropriate (median 9, DI <1) and feasible (median ≥7, DI <1) |  |  |
|  | 130 | Weekly review about discharge must be conducted by approved clinician or nominated deputy in relation to discharge |
| Appropriate (median ≥7, DI <1) and feasible (median ≥7, DI <1) |  |  |
|  | N/A | No statements ranked in this category |
| Appropriate (median ≥7, DI <1) but with uncertain feasibility (median ≤6.5 ≥3.5, DI <1) |  |  |
|  | 149 | DSR could happen within other daily meetings i.e. a tick-box or question in meeting 'has discharge been considered for this patient?' |
|  | 154 | DSR should happen at a set time in the day (i.e. shift handover, when junior doctor comes on ward, board review) |
|  | k4 | K4.  Every patient has a Daily Senior Review (DSR) before midday by a clinician able to make management and discharge decisions |
| Appropriate (median ≥7, DI <1) but infeasible (median ≤3, DI <1) |  |  |
|  | N/A | No statements ranked in this category |
| Uncertain appropriateness (median ≤6.5, ≥3.5, DI <1) but feasible |  |  |
|  | N/A | No statements ranked in this category |
| Uncertain appropriateness (median ≤6.5, ≥3.5, DI <1) with uncertain feasibility (median ≤6.5, ≥3.5, DI <1) |  |  |
|  | 133 | If DSR is not conducted by a consultant it should be supervised by a consultant |
|  | 134 | If DSR is not conducted by a consultant it should be reviewed by a consultant |
|  | 135 | If the member of staff conducting the DSR is not a consultant they should have received specialist training |
|  | 145 | DSR about discharge must always be communicated to carer |
|  | 146 | DSR about discharge must always be communicated to carer verbally |
|  | 147 | DSR about discharge can happen in absence of the carer in writing |
|  | 150 | DSR (regarding discharge) should be done as part of a stand alone meeting |
|  | 151 | DSR must happen before midday |
|  | 152 | DSR must happen before the end of the working day (5pm) |
|  | 153 | DSR can happen at any point during the day |
| Inappropriate (median ≤3, DI <1) and infeasible (median <3, DI <1). |  |  |
|  | 130 | DSR about discharge must be conducted by a consultant |
|  | 131 | DSR about discharge must be conducted by a senior nurse or higher |
|  | 137 | Older patients must receive a DSR about discharge |

**Table 5. Early flow: statements grouped by ranking**

| **Ranking score** | **Statement number** | **Statement** |
| --- | --- | --- |
| Appropriate (median 9, DI <1) and feasible (median 9, DI <1) |  |  |
|  | N/A | No statements ranked in this category |
| Appropriate (median 9, DI <1) and feasible (median ≥7, DI <1) |  |  |
|  | N/A | No statements ranked in this category |
| Appropriate (median ≥7, DI <1) and feasible (median ≥7, DI <1) |  |  |
|  | N/A | No statements ranked in this category |
| Appropriate (median ≥7, DI <1) but with uncertain feasibility (median ≤6.5 ≥3.5, DI <1) |  |  |
|  | k5 | Patient Flow must commence at the earliest opportunity from assessment units to inpatient wards. |
| Appropriate (median ≥7, DI <1) but infeasible (median ≤3, DI <1) |  |  |
|  | N/A | No statements ranked in this category |
| Uncertain appropriateness (median ≤6.5, ≥3.5, DI <1) but feasible |  |  |
|  | N/A | No statements ranked in this category |
| Uncertain appropriateness (median ≤6.5, ≥3.5, DI <1) with uncertain feasibility (median ≤6.5, ≥3.5, DI <1) |  |  |
|  | 155 | EF targets must be set to ensure that the first patient arrives onto the ward by 10am |
|  | 157 | Staff should have patients on the ward as early in the day as possible to enable EF, but not as an official target or audited. |
|  | 170 | People bringing a patient on the ward should notify the ward when they leave to enable EF |
|  | 171 | Newly admitted patients should be introduced to the ward staff by the referring professional to enable EF |
|  | k6 | Wards that routinely receive patients from assessment units will ensure the first patient arrives on the ward by 10am |
| Inappropriate (median ≤3, DI <1) and infeasible (median <3, DI <1). |  |  |
|  | 164 | EF targets must be set to ensure that the first patient arrives onto the ward by 11am |
|  | 165 | EF targets must be set to ensure that the first patient arrives onto the ward by 12pm |
|  | 166 | EF targets must be set to ensure that the first patient arrives onto the ward by 1pm |
|  | 167 | EF targets must be set and audited to ensure that the first patient arrives on the ward by 11am |
|  | 168 | EF targets must be set and audited to ensure that the first patient arrives on the ward by 12pm |
|  | 169 | EF targets must be set and audited to ensure that the first patient arrives on the ward by 1pm |

**Table 6. Multi-disciplinary team discharge meeting: statements grouped by ranking**

| **Ranking score** | **Statement number** | **Statement** |
| --- | --- | --- |
| Appropriate (median 9, DI <1) and feasible (median 9, DI <1) |  |  |
|  | N/A | No statements ranked in this category |
| Appropriate (median 9, DI <1) and feasible (median ≥7, DI <1) |  |  |
|  | N/A | No statements ranked in this category |
| Appropriate (median ≥7, DI <1) and feasible (median ≥7, DI <1) |  |  |
|  | 172 | An MDT meeting to discuss discharge must be conducted for every patient who has been on the ward for longer than 7 days |
|  | 175 | An MDT meeting to discuss discharge must be conducted at the agreed time (7/14/21 days) for all patients |
|  | 184 | An MDT meeting to discuss discharge of patients can be combined into existing MDT meetings |
|  | 185 | An MDT meeting to discuss discharge of patients must include the patient |
|  | 186 | An MDT meeting to discuss discharge of patients must include the patient if they wish to be involved (i.e. no social anxiety) |
|  | 187 | An MDT meeting to discuss discharge of patients can include carers where agreed by patients |
|  | 189 | An MDT meeting to discuss discharge of patients must be face-to-face |
|  | 190 | An MDT meeting to discuss discharge of patients can use remote technology to bring together professionals |
|  | 191 | An MDT meeting to discuss discharge of patients must include professionals from relevant community organisations |
|  | 193 | An MDT meeting to discuss discharge of patients must include professionals from relevant community organisations for all patients where applicable |
|  | 201 | Consultant ward-based psychiatrists must attend all discharge MDT meetings |
|  | 209 | Care co-ordinator must attend all discharge MDT meetings |
|  | 219 | Consultant ward-based psychiatrists must attend final discharge MDT meeting |
|  | 227 | Care co-ordinator must attend final discharge MDT meeting |
|  | 243 | MDT discharge meetings can be technology assisted |
|  | 244 | Informal carers can attend meetings using technology/teleconferencing/videoconferencing |
|  | 245 | Meetings between patients on the ward and professionals in the community can be technology assisted |
|  | 246 | Meetings between ward staff and professionals from external agencies (social work, CMHT etc.) can be technology assisted |
| Appropriate (median ≥7, DI <1) but with uncertain feasibility (median ≤6.5 ≥3.5, DI <1) |  |  |
|  | 192 | An MDT meeting to discuss discharge of patients must include professionals from relevant community organisations if they are known to services and a history of readmission |
|  | 204 | Ward-based band 5 nurses must attend all discharge MDT meetings |
|  | 214 | Community mental health nurses must attend all discharge MDT meetings |
|  | 220 | Ward-based Speciality training ST1 up to consultant doctors must attend final discharge MDT meeting |
|  | 225 | Ward-based pharmacists must attend final discharge MDT meeting |
|  | 231 | Crisis team (or equivalent) professionals must attend final discharge MDT meeting |
|  | 232 | Community mental health nurses must attend final discharge MDT meeting |
|  | 250 | Each person must have their own device |
|  | 256 | Patient's advocate must attend all discharge MDT meetings |
|  | k7 | A systematic Multi-disciplinary Discharge Team (MDT) review is held for patients with extended length of stay with a clear 'home first' mind-set |
| Appropriate (median ≥7, DI <1) but infeasible (median ≤3, DI <1) |  |  |
|  | N/A | No statements ranked in this category |
| Uncertain appropriateness (median ≤6.5, ≥3.5, DI <1) but feasible |  |  |
|  | 196 | An MDT meeting to discuss discharge of patients must include a wider range of non-ward based professionals when discharge is less than 14 days away |
|  | 197 | An MDT meeting to discuss discharge of patients must include a wider range of non-ward based professionals when discharge is less than 7 days away |
|  | 248 | Meetings between two or more ward-based staff can be technology assisted |
|  | 249 | Meetings between ward staff and patients can be technology assisted |
| Uncertain appropriateness (median ≤6.5, ≥3.5, DI <1) with uncertain feasibility (median ≤6.5, ≥3.5, DI <1) |  |  |
|  | 173 | An MDT meeting to discuss discharge must be conducted for every patient who has been on the ward for longer than 14 days |
|  | 174 | An MDT meeting to discuss discharge must be conducted for every patient who has been on the ward for longer than 21 days |
|  | 176 | An MDT meeting to discuss discharge must be conducted at the agreed time for older patients |
|  | 177 | An MDT meeting to discuss discharge must be conducted at the agreed time for voluntary patients |
|  | 178 | An MDT meeting to discuss discharge must be conducted at the agreed time for involuntary patients |
|  | 180 | After the initial meeting an MDT meeting to discuss discharge must continue to be conducted on a fortnightly basis |
|  | 181 | After the initial meeting an MDT meeting to discuss discharge must continue to be conducted on a weekly basis |
|  | 182 | After the initial meeting an MDT meeting to discuss discharge must continue to be conducted on a monthly basis |
|  | 188 | An MDT meeting to discuss discharge of patients must include carers |
|  | 198 | An MDT meeting to discuss discharge of patients must include a wider range of non-ward based professionals when discharge is less than 72 hours away |
|  | 199 | An MDT meeting to discuss discharge of patients must include a wider range of non-ward based professionals when discharge is less than 48 hours away |
|  | 200 | An MDT meeting to discuss discharge of patients must include a wider range of non-ward based professionals when discharge is less than 24 hours away |
|  | 202 | Ward-based Speciality training ST1 up to consultant   must attend all discharge MDT meetings |
|  | 203 | Ward-based Core Training 1-3 doctors must attend all discharge MDT meetings |
|  | 205 | Ward-based senior nurses must attend all discharge MDT meetings |
|  | 206 | Ward-based healthcare assistants/nursing assistants must attend all discharge MDT meetings |
|  | 207 | Ward-based pharmacists must attend all discharge MDT meetings |
|  | 208 | Community pharmacists must attend all discharge MDT meetings |
|  | 211 | Social work professionals must attend all discharge MDT meetings |
|  | 212 | Housing professionals must attend all discharge MDT meetings |
|  | 213 | Crisis team (or equivalent) professionals must attend all discharge MDT meetings |
|  | 215 | Community mental health doctors must attend all discharge MDT meetings |
|  | 216 | Ward-based pharmacy technicians must attend all discharge MDT meetings |
|  | 217 | Ward-based occupational therapists must attend all discharge MDT meetings |
|  | 218 | Community occupational therapists must attend all discharge MDT meetings |
|  | 221 | Ward-based Core Training 1-3 doctors must attend final discharge MDT meeting |
|  | 222 | Ward-based band 5 nurses must attend final discharge MDT meeting |
|  | 223 | Ward-based senior nurses must attend final discharge MDT meeting |
|  | 224 | Ward-based healthcare assistants/nursing assistants must attend final discharge MDT meeting |
|  | 226 | Community pharmacists must attend final discharge MDT meeting |
|  | 228 | Primary care professionals must attend final discharge MDT meeting |
|  | 229 | Social work professionals must attend final discharge MDT meeting |
|  | 230 | Housing professionals must attend final discharge MDT meeting |
|  | 233 | Community mental health doctors must attend final discharge MDT meeting |
|  | 234 | Ward-based pharmacy technicians must attend final discharge MDT meeting |
|  | 235 | Ward-based occupational therapists must attend final discharge MDT meeting |
|  | 236 | Community occupational therapists must attend discharge MDT meeting |
|  | 241 | Community carers must attend final discharge MDT meeting |
|  | 242 | Community carers must attend all discharge MDT meetings |
|  | 247 | Meetings between ward staff and professionals from external agencies (social work, CMHT etc.) must be technology assisted |
|  | 251 | Groups can share a communal device |
|  | 253 | Telemedicine and online video calls are an appropriate method of discussion of discharge by MDT |
|  | 254 | Zoom is an appropriate method of discussion of discharge |
|  | 255 | Skype is an appropriate method of discussion of discharge |
| Inappropriate (median ≤3, DI <1) and infeasible (median <3, DI <1). |  |  |
|  | 238 | Bed managers must attend all discharge MDT meetings |
|  | 239 | General practitioner must attend final discharge MDT meeting |
|  | 240 | General Practitioner must attend all discharge MDT meetings |

**Table 7. Multi-agency discharge team: statements grouped by ranking**

| **Ranking score** | **Statement number** | **Statement** |
| --- | --- | --- |
| Appropriate (median 9, DI <1) and feasible (median 9, DI <1) |  |  |
|  | N/A | No statements ranked in this category |
|  | N/A | No statements ranked in this category |
| Appropriate (median 9, DI <1) and feasible (median ≥7, DI <1) |  |  |
|  | N/A | No statements ranked in this category |
| Appropriate (median ≥7, DI <1) and feasible (median ≥7, DI <1) |  |  |
|  | 260 | MADT should meet in person |
|  | 261 | MADT should meet in person with the option for people to join virtually |
|  | 262 | MADT should meet at the same time each week |
|  | 266 | Community mental health teams should be part of MADT |
|  | 267 | A crisis team member should be part of the MADT |
|  | 272 | A ward-based psychiatrist should be part of the MADT |
|  | 274 | A ward-based nurse should be part of the MADT |
|  | 276 | A ward-based occupational therapist should be part of the MADT |
|  | 277 | A Care co-ordinator should be part of the MADT |
|  | 283 | Any patient can be discussed by the MADT |
|  | 288 | MADT discharge meetings can be technology assisted |
| Appropriate (median ≥7, DI <1) but with uncertain feasibility (median ≤6.5 ≥3.5, DI <1) |  |  |
|  | 257 | MADT should meet each week |
|  | 275 | A ward-based clinical psychologist should be part of the MADT |
|  | k8 | K8. Multi-Agency Discharge Teams (MADT) is established in the Trust for every eligible patient as early as possible, with representatives from several community agencies (ward, police, ambulance, housing, substance misuse, social services etc.) |
| Appropriate (median ≥7, DI <1) but infeasible (median ≤3, DI <1) |  |  |
|  | 291 | Zoom is an appropriate method of discussion of discharge |
|  | 296 | Carers can attend the meeting remotely |
| Uncertain appropriateness (median ≤6.5, ≥3.5, DI <1) but feasible |  |  |
|  | N/A | No statements ranked in this category |
| Uncertain appropriateness (median ≤6.5, ≥3.5, DI <1) with uncertain feasibility (median ≤6.5, ≥3.5, DI <1) |  |  |
|  | 258 | MADT should meet each fortnight |
|  | 259 | MADT should meet as and when needed depending on patient admissions and discharges |
|  | 263 | MADT should meet virtually |
|  | 265 | Representatives from housing organisations should be part of the MADT |
|  | 268 | A liaison team member should be part of the MADT |
|  | 269 | A homeless organisation representative should be part of the MADT |
|  | 270 | A bed manager should be part of the MADT |
|  | 271 | An inpatient service manager should be part of the MADT |
|  | 273 | A ward-based pharmacist should be part of the MADT |
|  | 278 | A social worker should be part of the MADT |
|  | 279 | The team should be named 'transitions team' |
|  | 280 | The team should be named 'mental health inter-agency team' |
|  | 282 | Only complex cases involved with multiple services will be discussed by the MADT |
|  | 286 | Patients must receive pre-determined individual time slots to be discussed by the MADT |
|  | 287 | Patients should have an option join the MADT discussion in their allocated individual time slots |
|  | 289 | Telephone is an appropriate method of discussion of discharge |
|  | 290 | Telemedicine and online video calls are an appropriate method of discussion of discharge by MDT |
|  | 292 | Skype is an appropriate method of discussion of discharge |
|  | 293 | Each person must have their own device |
|  | 294 | Groups can share a communal device |
|  | 295 | Carers should be invited to the MADT |
| Inappropriate (median ≤3, DI <1) and infeasible (median <3, DI <1). |  |  |
|  | N/A | No statements ranked in this category |

**Table 8. Patient written discharge plan: statements grouped by ranking**

| **Ranking score** | **Statement number** | **Statement** |
| --- | --- | --- |
| Appropriate (median 9, DI <1) and feasible (median 9, DI <1) |  |  |
|  | N/A | No statements ranked in this category |
| Appropriate (median 9, DI <1) and feasible (median ≥7, DI <1) |  |  |
|  | 340 | PWDP must include a contact phone number for help post-discharge (i.e. if I have problems I must call:) |
| Appropriate (median ≥7, DI <1) and feasible (median ≥7, DI <1) |  |  |
|  | 297 | Every patient must be given the opportunity to independently write their own PWDP upon admission |
|  | 298 | The PWDP must replace an existing discharge planning documentation |
|  | 299 | The PWDP must be in addition to the discharge planning documentation |
|  | 300 | The items that are included in a PWDP are determined by the patient, using a number of prompts on a proforma |
|  | 301 | PWDPs must be created with assistance from a healthcare professional (and written by the professional if the patient does not want to independently write the plan) |
|  | 305 | PWDPs must be co-created with a persons named staff member/key worker |
|  | 306 | PWDPs must be sent to community team/care co-ordinator |
|  | 307 | PWDPs must be sent to social workers (if involved) |
|  | 308 | PWDP must be sent to primary care |
|  | 312 | The PWDP must be held by the patient |
|  | 313 | The PWDP must be recorded in existing acute systems (i.e. scanned) |
|  | 314 | The PWDP must be semi-structured |
|  | 318 | The PWDP must be introduced to the patient within 7 days of admission |
|  | 326 | Patients must be encouraged to ask questions and record answers about the things that matter most to them at PWDP |
|  | 329 | Carers must be involved in the development of the PWDP, if the patient agrees |
|  | 330 | The PWDP must include a section for carers |
|  | 336 | PWDP must include patients name |
|  | 337 | PWDP must include patients estimated date of discharge |
|  | 338 | PWDP must include patients actual date of discharge |
|  | 339 | PWDP must include a contact name for help post-discharge (i.e. if I have problems I must call:) |
|  | 342 | PWDP must include a summary of medical problem/diagnosis in plain English |
|  | 343 | PWDP must include prompts about medication allergies |
|  | 345 | PWDP must include prompts about activities that are good for the patient (i.e. what activities are good for me?) |
|  | 346 | PWDP must include prompts about nutritional advice (i.e. what must I eat?) |
|  | 348 | PWDP must include a table of medications |
|  | 349 | PWDP must include numerous medication tables split by day (morning, afternoon, evening, bedtime) |
|  | 350 | Medication table within PWDR must include medicine name |
|  | 352 | Medication table within PWDR must include medicine generic name |
|  | 353 | Medication table within PWDR must include rationale for medication (i.e. why am I taking this medication) |
|  | 354 | Medication table within PWDR must include dosage (i.e. how much must I take?) |
|  | 355 | Medication table within PWDR must include dosage in plain English (i.e. how much must I take?) |
|  | 356 | Medication table within PWDR must include instructions about medication methods (i.e. how do I take medication?) |
|  | 357 | PWDP must include information about other medications for non-mental health ailments (i.e. what other medications can I take) |
|  | 360 | PWDP must include information about any other medications that are relevant to the patient, with a blank space (i.e. if I need medicine for _______) |
|  | 361 | PWDP must include medicine name and amount for non-mental health medication |
|  | 362 | PWDP must include instructions for taking for non-mental health medication |
|  | 363 | PWDP must include information about next appointment |
|  | 364 | PWDP must include date of next appointment |
|  | 365 | PWDP must include time of next appointment |
|  | 366 | PWDP must include clinicians name (for next appointment) |
|  | 367 | PWDP must include location and address of next appointment |
|  | 368 | PWDP must include reason for next appointment |
|  | 369 | PWDP must include contact number for next appointment |
|  | 374 | PWDP must include 'feeling stressed' as a prompt for questions to ask at next appointment |
|  | 375 | PWDP must include 'feelings' as a prompt for questions to ask at next appointment |
|  | 376 | PWDP must include 'other concerns' as a prompt for questions to ask at next appointment |
|  | 381 | PWDP must include notes section |
|  | 382 | Patient should chose which professional they would like to create the PWDP with |
|  | k9 | K9. A Patient Written Discharge Plan (PWDP) developed for each patient |
| Appropriate (median ≥7, DI <1) but with uncertain feasibility (median ≤6.5 ≥3.5, DI <1) |  |  |
|  | 344 | PWDP must include prompts location of community pharmacy |
| Appropriate (median ≥7, DI <1) but infeasible (median ≤3, DI <1) |  |  |
|  | N/A | No statements ranked in this category |
| Uncertain appropriateness (median ≤6.5, ≥3.5, DI <1) but feasible |  |  |
|  | 309 | An abbreviated version of the PWDP must be sent to primary care |
|  | 310 | An abbreviated version of the PWDP must be sent to community care teams |
|  | 311 | An abbreviated version of the PWDP must be sent to social services (if involved) |
|  | 319 | The PWDP must be introduced to the patient within 14 days of admission |
|  | 323 | The PWDP must be introduced to the patient 72 hours before discharge |
|  | 324 | The PWDP must be introduced to the patient 7 days before discharge |
|  | 332 | Patients must take the completed PWDP home |
|  | 341 | PWDP must include a reminder in bold on the front page to take it to medical appointments |
|  | 347 | PWDP must include prompts about food or activities to avoid |
|  | 351 | Medication table within PWDR must include medicine brand name |
|  | 358 | PWDP must include information about medications for headaches |
|  | 370 | PWDP must include prompts for questions to ask at next appointment |
|  | 371 | PWDP must include 'medicine' as a prompt for questions to ask at next appointment |
|  | 372 | PWDP must include 'test results' as a prompt for questions to ask at next appointment |
|  | 373 | PWDP must include 'pain' as a prompt for questions to ask at next appointment |
| Uncertain appropriateness (median ≤6.5, ≥3.5, DI <1) with uncertain feasibility (median ≤6.5, ≥3.5, DI <1) |  |  |
|  | 302 | PWDPs must be co-created with a doctor |
|  | 303 | PWDPs must be co-created with a nurse |
|  | 304 | PWDPs must be co-created with a healthcare assistant/nursing assistant |
|  | 315 | The PWDP must be completely structured |
|  | 316 | The PWDP mustn't be structured |
|  | 320 | The PWDP must be introduced to the patient within 24 hours of admission |
|  | 325 | The PWDP must be introduced to the patient 14 days before discharge |
|  | 327 | Carers must be involved in the development of the PWDP |
|  | 328 | Carers must be informed about the information in the PWDP |
|  | 331 | Patients must talk with a member of staff about each of the items included in PWDP |
|  | 359 | PWDP must include information about medications for smoking cessation |
|  | 377 | PWDP must include information sections about the next two appointments |
|  | 378 | PWDP must include information sections about the next three appointments |
|  | 379 | PWDP must include information sections about the next four appointments |
|  | 380 | PWDP must include information sections about the next five appointments |
| Inappropriate (median ≤3, DI <1) and infeasible (median <3, DI <1). |  |  |
|  | 334 | Patients must take the PWDP to all appointments with general practitioners |
|  | 335 | Patients must take the PWDP to all appointments with all healthcare professionals |

**Table 9. Primary care discharge summary: statements grouped by ranking**

| **Ranking score** | **Statement number** | **Statement** |
| --- | --- | --- |
| Appropriate (median 9, DI <1) and feasible (median 9, DI <1) |  |  |
|  | N/A | No statements ranked in this category |
| Appropriate (median 9, DI <1) and feasible (median ≥7, DI <1) |  |  |
|  | 391 | Patient name must be included on the PCDS |
|  | 392 | Patient preferred name must be included on the PCDS |
|  | 393 | Patient date of birth must be included on the PCDS |
|  | 395 | Patient NHS number must be included on the PCDS |
|  | 416 | Reason for admission must be included in the PCDS (i.e. he health problems and issues experienced by the patient that prompted the decision to admit to hospital) |
|  | 421 | Discharge details must be included in the PCDS |
|  | 425 | Date and time of discharge must be included in the PCDS |
|  | 445 | Consent relating to child must be included in the PCDS (i.e. record of person with parental responsibility or appointed guardian where child lacks competency) |
|  | 450 | Safeguarding issues must be included in the PCDS (i.e.. any legal matters relating to safeguarding of a vulnerable child or adult, e.g., child protection plan, protection of vulnerable adult.) |
|  | 452 | Risk to self must be included in the PCDS (i.e. any risk the patient poses to themselves- suicide, self-harm etc.) |
|  | 458 | Person completing record must be included in the PCDS |
|  | 462 | Date and time of completion of PCDS must be included |
|  | 477 | Medication name must be included in the PCDS |
|  | 479 | Medication quantity supplied on discharge must be included in the PCDS |
|  | 483 | Dose directions description must be included in the PCDS (A single plain text phrase describing the entire medication dosage and administration directions, including dose quantity and medication frequency) |
|  | 484 | Dose amount description must be included in the PCDS (A plain text description of medication single dose amount, e.g. 30mg or 2 tabs) |
|  | 485 | Dose timing description must be included in the PCDS (A plain text description of medication dose frequency e.g. Twice a day, at 8am, 2pm and 10pm) |
|  | 486 | Structured dose direction must be included in the PCDS (Recommendation of the time period for which the medication should be continued, including direction not to discontinue) |
|  | 512 | Description of allergies or adverse reactions must be included in the PCDS |
| Appropriate (median ≥7, DI <1) and feasible (median ≥7, DI <1) |  |  |
|  | 383 | PCDS must have clear action points |
|  | 384 | Clear action points should be highlighted at the start of the PCDS |
|  | 385 | Clear action points should be highlighted at the end of the PCDS |
|  | 387 | Expectation of continuation or amendments to psychotropic medication in community must be clearly highlighted |
|  | 388 | Action points for GPs must be included on the PCDS |
|  | 389 | Action points for GPs must be highlighted on the PCDS |
|  | 390 | Discharge summaries must use a standard proforma |
|  | 394 | Patient gender must be included on the PCDS |
|  | 396 | Patient other identifier must be included on the PCDS (i.e. Community Health Index- Scotland) |
|  | 397 | Patient address must be included on the PCDS |
|  | 399 | Patient telephone number must be included on the PCDS |
|  | 400 | Relevant contacts must be included on the PCDS (next of kin, emergency contact) |
|  | 401 | Details of GP practice where patient is registered must be included on the PCDS |
|  | 404 | Referrer details must be included on the PCDS (details of individual or team who referred the patient for that admission) |
|  | 409 | Individual requirements must be included in the PCDS (i.e. communication, cognitive, mobility needs) |
|  | 415 | Admission details must be included in the PCDS |
|  | 417 | Admission method must be included in the PCDS (i.e. how the patient was admitted to hospital. for example: involuntary, through emergency services, transfer etc.) |
|  | 418 | Admission method must be included in the PCDS (How the patient was admitted to hospital. For example: elective, emergency, maternity, transfer etc.) |
|  | 419 | Date/time of admission must be included in the PCDS |
|  | 420 | Source/location of admission must be included in the PCDS (i.e. where the patient was immediately prior to admission, e.g. usual place of residence, temporary place of residence, penal establishment.) |
|  | 422 | Discharging consultant must be included in the PCDS |
|  | 423 | Discharging speciality/department must be included in the PCDS |
|  | 424 | Discharge location must be included in the PCDS (the ward or unit the patient was in immediately 'priori' to discharge) |
|  | 426 | Method of discharge from hospital must be included in the PCDS (i.e. patient discharged on clinical advice or with clinical consent; patient discharged him/herself or was discharged by a relative or advocate; patient died) |
|  | 427 | Discharge destination cluster must be included in the PCDS (i.e. the destination of the patient on discharge) |
|  | 428 | Diagnosis must be included in the PCDS (a list of diagnoses) |
|  | 429 | Diagnosis name must be included in the PCDS (confirmed diagnosis or symptom, active diagnosis being treated) |
|  | 430 | Diagnosis stage must be included in the PCDS where relevant (i.e. does the patient have a diagnosis, how long have they had it) |
|  | 431 | Diagnosis comments must be included in the PCDS (supporting text covering diagnosis) |
|  | 432 | Procedures must be included in the PCDS (details of any procedures performed) |
|  | 436 | Complications related to procedure must be included in the PCDS |
|  | 437 | Specific anaesthesia issues related to procedure must be included in the PCDS (i.e. details of any adverse reaction to any anaesthetic agents including local anaesthesia. Problematic intubation, transfusion reaction, etc.) |
|  | 438 | Comments in relation to procedure must be included in the PCDS |
|  | 439 | Clinical summary must be included in the PCDS (Summary of the encounter. Where possible, very brief. This may include interpretation of findings and results; differential diagnoses, opinion and specific actions) |
|  | 440 | Investigation results must be included in the PCDS (For each investigation, the result of the investigation (this includes the result value, with unit of observation and reference interval where applicable and date, and plans for acting upon investigation results.) |
|  | 443 | Consent for treatment record must be included in the PCDS (Whether consent has been obtained for the treatment. May include where record of consent is located or record of consent.) |
|  | 444 | Consent for information sharing must be included in the PCDS |
|  | 446 | Mental capacity assessment must be included in the PCDS |
|  | 447 | Advance decision to refuse treatment (ADRT) must be included in the PCDS |
|  | 448 | Lasting power of attorney for personal welfare or court-appointed deputy (or equivalent) must be included in PCDS |
|  | 451 | Safety alerts must be included in the PCDS (i.e. details of any risks the patient poses to themselves or others.) |
|  | 453 | Risk to others must be included in the PCDS (i.e. any risk to caring professionals or others) |
|  | 454 | Risk from others must be included in the PCDS (i.e. details of where an adult or child is at risk from an identified person e.g. family member etc.) |
|  | 455 | Description of patient and carer concerns, expectations and wishes must be included in the PCDS |
|  | 456 | Patient and carer concerns, expectations and wishes must be included in the PCDS |
|  | 457 | Advance statement must be included in the PCDS (Written requests and preferences made by a person with capacity conveying their wishes, beliefs and values for their future care must they lose capacity) |
|  | 459 | Name, role and organisation of person completing record must be included in the PCDS |
|  | 460 | Grade of person completing PCDS must be included |
|  | 461 | Speciality of person completing PCDS must be included |
|  | 463 | Professional identifier of person completing PCDS must be included |
|  | 464 | Plan and requested actions must be included in the PCDS |
|  | 465 | Actions for healthcare professionals must be included in the PCDS (including planned investigations, procedures and treatment for a patient’s identified conditions and priorities. For each action the following must be identified: outcome expectations, including patient’s expectations) |
|  | 466 | Actions for patients or their carer must be included in the PCDS (For each action the following must be identified: outcome expectations, including patient’s expectations) |
|  | 467 | Indications of whether the plan has been agreed with patient or legitimate patient representative (e.g. whether agreed the entire plan or individual aspects of treatment, expected outcomes, risks and alternative treatments) |
|  | 468 | Investigations requested must be included in the PCDS (i.e. name or description and date) |
|  | 469 | Procedures requested must be included in the PCDS (i.e. name or description and date) |
|  | 470 | Distribution list must be included in the PCDS (a list of other individuals to receive a copy of this communication) |
|  | 471 | Name, role and organisation of person to receive a copy of this documentation must be included in the PCDS |
|  | 473 | Team of person to receive a copy of this documentation must be included in the PCDS |
|  | 475 | Medications and Medical devices must be included in the PCDS (i.e. The details of and instructions for medications and medical equipment the patient is using.) |
|  | 476 | Medication items must be included in the PCDS (details of continuation / addition / amendment of admission medications) |
|  | 478 | Medication form must be included in the PCDS (e.g. capsules, tablets, liquid) |
|  | 480 | Medication route must be included in the PCDS (Medication administration description (oral, IM, IV, etc.): may include method of administration, (e.g., by infusion, via nebuliser, via NG tube). |
|  | 481 | Medication site must be included in the PCDS (anatomical site) |
|  | 482 | Medication technique must be included in the PCDS (technique or method by which medication is administered) |
|  | 487 | Structured dose amount must be included in the PCDS (a structural representation of dose amount. Comment: e.g. 20mg or 2 tablets) |
|  | 488 | Structured dose timing must be included in the PCDS (A structural, computable representation of dose timing and maximum dose) |
|  | 489 | Dose direction duration must be included in the PCDS (Recommendation of the time period for which the medication should be continued, including direction not to discontinue) |
|  | 490 | Additional instructions regarding medication should be included in the PCDS (Additional multiple dosage or administration instructions as plain text. This may include guidance to the prescriber, patient or person administering the medication, e.g. omit morning dose on day of procedure or dispense weekly) |
|  | 491 | Course details must be included in the PCDS (details pf overall course of medication) |
|  | 492 | Course status must be included in the PCDS (The status of this prescription in an ambulatory (outpatient/GP/community) context- dose/titration regimes) |
|  | 493 | Course start date and time must be included in the PCDS |
|  | 494 | Course end date and time must be included in the PCDS |
|  | 495 | Indication must be included in the PCDS (reason for medication being prescribed) |
|  | 497 | Comment/recommendation must be included in the PCDS (suggestion about duration and/or review, on-going monitoring requirements, advice on starting, discontinuing or changing medication |
|  | 498 | Medication change summary must be included in the PCDS (changes made to medication since admission) |
|  | 499 | The nature/status of any medication change since admission must be include in the PCDS |
|  | 500 | The indication of any medication change since admission must be include in the PCDS (reason for change) |
|  | 501 | The date of latest medication change since admission must be include in the PCDS (including addition or amendment) |
|  | 502 | Description of medication change must be included in the PCDS (Where a change is made to the medication- i.e. one drug stopped, another started, or dose, frequency or route is change) |
|  | 503 | Total dose daily quantity must be included in the PCDS (Total daily dose of medication) |
|  | 504 | Medical devices must be included in PCDS |
|  | 505 | Medication discontinuation must be included in the PCDS |
|  | 506 | Name of discontinuatied medication must be included in the PCDS |
|  | 507 | Status of discontinued medication must be included in the PCDS |
|  | 508 | Indication of discontinued medication must be included in the PCDS (reason why) |
|  | 511 | Comments about discontinued medication must be included in the PCDS |
|  | 513 | Causative agent of allergies or adverse reactions must be included in the PCDS (I.e. food, drug or substances) |
|  | 514 | Reaction details must be included in the PCDS |
|  | 515 | Description, severity and certainty of reaction must be included in the PCDS |
|  | 516 | Date of reaction must be included in the PCDS |
|  | 518 | Type of reaction must be included in the PCDS |
|  | 519 | Evidence of reaction must be included in the PCDS (results of investigations) |
|  | 521 | PCDS sent to GP must include a summary of patient written discharge plan |
|  | 522 | PCDS sent to GP must include full patient written discharge plan |
|  | 528 | Discharge summary should also be sent to substance misuse specialist (if identified) |
|  | 529 | Known use of illicit substances in the community must be included in the PCDS |
|  | 530 | Alcohol intake must be included in the PCDS |
|  | 531 | Smoking status must be included in the PCDS |
|  | 532 | PCDS must specify if patient is in receipt of multi-compartment compliance aid (dosette box) (MDS) |
|  | 534 | PCDS must specify whether MDS is self-filled by patient or family or filled by pharmacy |
|  | 535 | PCDS must specify (and differentiate between) whether there is a new MDS or existing MDS that needs to be continued |
|  | 536 | PCDS must capture if MDS is discontinued during admission and reasons why this in not continued post-discharge inappropriately |
|  | 537 | PCDS must specify whether patient is to self-administer medication or if another (e.g. carer, family member) is to do this |
|  | 538 | PCDS must specify whether patient has been self-administering medication during admission |
|  | 539 | PCDS must include medication taking support required (i.e. large print labels, easy open bottles, swallowing difficulties, reminder aids, |
|  | 540 | PCDS must identify whether recording any potential medication risks is important (i.e. non-adherence, access by children to medication, risk of overdose requiring 7 day supplies) |
|  | 542 | PCDS should capture information provision about prescribed medication and medication changes prior to discharge from hospital |
|  | 544 | Substance misuse medications and contact details of managing substance misuse team and collection details (e.g. daily supervised or unsupervised consumption) must be included in PCDS |
|  | 546 | PCDS must include information capture regarding medication adherence with details (both pre-hospital adherence and adherence during stay) |
|  | 547 | Medication reconciliation conducted at admission must be used to support completion of PCDS |
|  | 548 | PCDS must include where further supplies of medication may be obtained as some may be obtained from hospital pharmacy (clozapine, depot clinic) rather than the GP. |
|  | 549 | Its important to capture both allergies and intolerances (which are not allergies) in the PCDS |
|  | 550 | Common side effects of medication must be included in PCDS |
|  | 551 | Medication currently prescribed to counter side effects must be included in PCDS |
|  | k10 | K10. Standardised and high quality Primary Care Discharge Summaries (PCDS) must be used |
| Appropriate (median ≥7, DI <1) but with uncertain feasibility (median ≤6.5 ≥3.5, DI <1) |  |  |
|  | 442 | Legal information must be included in the PCDS (i.e. legal information captured relating to patient care, such as consent to treatment and mental capacity) |
|  | 496 | Link to indication record must be included in the PCDS |
|  | 509 | Date of discontinuatied medication must be included in the PCDS |
|  | 517 | Comments about reaction must be included in the PCDS |
|  | 520 | Date reaction first experienced must be included in the PCDS |
|  | 523 | The healthcare professional that works with patient to develop patient written discharge plan, creates a summary to add to patient record which is sent to GP |
|  | 524 | The healthcare professional that works with patient to develop patient written discharge plan, creates a summary to add to patient record which is sent to GP |
|  | 525 | The doctor responsible for the PCDS, creates a summary paragraph of the key components of the patient written discharge plan |
|  | 533 | PCDS must specify which pharmacy supplies the multi-compartment compliance aid (dosette box) (MDS) |
| Appropriate (median ≥7, DI <1) but infeasible (median ≤3, DI <1) |  |  |
|  | N/A | No statements ranked in this category |
| Uncertain appropriateness (median ≤6.5, ≥3.5, DI <1) but feasible |  |  |
|  | 386 | PCDS must always be addressed to a GP |
|  | 403 | GP practice identifier must be included (i.e. ODS- Organisation Data Services) |
|  | 433 | Procedure name must be included in the PCDS (details of any procedures performed) |
|  | 434 | Procedure anatomical site must be included in the PCDS |
|  | 435 | Laterality of procedure must be included in the PCDS |
|  | 474 | Relationship to patient of person to receive a copy of this documentation must be included in the PCDS |
|  | 526 | Discharge summary should also be sent to practice nurse |
| Uncertain appropriateness (median ≤6.5, ≥3.5, DI <1) with uncertain feasibility (median ≤6.5, ≥3.5, DI <1) |  |  |
|  | 398 | Patient email address must be included on the PCDS |
|  | 402 | GP name must be included on PCDS (i.e. GP name) |
|  | 405 | Patients social context must be included in the PCDS (i.e. social setting in which the patient lives- household, occupational history, lifestyle factors) |
|  | 406 | Household composition must be included in the patient PCDS (i.e. lives alone, lives with family, lives with partner) |
|  | 407 | Occupational history must be included in the PCDS (i.e. current and/or previous relevant occupations of the patient/individuals) |
|  | 408 | Educational history must be included in the PCDS (relevant current or previous) |
|  | 410 | Individual requirements must only include information volunteered by the patient or carer |
|  | 411 | Individual requirements can also include information known locally |
|  | 412 | Participation in research must be included in the PCDS (details of research studies participated in) |
|  | 413 | Name of the research study/trial must be included in the PCDS (details of research studies participated in) |
|  | 414 | Name of the research drug/intervention must be included in the PCDS (details of research studies participated in) |
|  | 441 | Description of any assessment scales used must be included in the PCDS |
|  | 449 | Organ and tissue donation must be included in the PCDS (i.e.. whether the person has given consent for organ and/or tissue donation or opted out of automatic donation where applicable.) |
|  | 472 | Grade of person to receive a copy of this documentation must be included in the PCDS |
|  | 510 | Description of discontinuatied medication must be included in the PCDS |
|  | 527 | Discharge summary should also be sent to practice pharmacist |
|  | 541 | PCDS should include whether patient needs to recieve information from primary care provider (community pharmacy or general practice) about prescribed medication. |
|  | 543 | All patients should be refered to a named community pharmacist (who receives a copy of the discharge summary) |
|  | 545 | PCDS must include a pharmacy specific comments section |
| Inappropriate (median ≤3, DI <1) and infeasible (median <3, DI <1). |  |  |
|  | N/A | No statements ranked in this category |

**Table 10. Social information capture: statements grouped by ranking**

| **Ranking score** | **Statement number** | **Statement** |
| --- | --- | --- |
| Appropriate (median 9, DI <1) and feasible (median 9, DI <1) |  |  |
|  | 571 | Every patient's safeguarding status must be captured in the patient record at admission |
| Appropriate (median 9, DI <1) and feasible (median ≥7, DI <1) |  |  |
|  | 552 | Every patient's accommodation status must be captured in the patient record at admission |
|  | 607 | Every patient's physical healthcare needs must be captured in the patient record at admission |
|  | 626 | Every patient's care giving responsibilities must be captured in the patient record at admission |
|  | 634 | Details of patient's preferences about communication with informal carer must be captured at admission |
|  | 636 | Every patient's involvement with other services (i.e. police, drug and alcohol) must be captured in the patient record at admission |
|  | 648 | Every patients General Practitioner details must be captured upon admission |
| Appropriate (median ≥7, DI <1) and feasible (median ≥7, DI <1) |  |  |
|  | 558 | If potential accommodation issues are highlighted at admission addressing this must be highlighted as action points for a specified member of staff within a specified time frame |
|  | 559 | Every patient's accommodation status must be requested from the referring/admitting professional upon admission (social worker, CMHT, crisis) |
|  | 560 | Every patient's accommodation status must be requested from the patient themselves upon admission (if not already in clinical notes from professionals) |
|  | 564 | If a potential accommodation problem is identified at admission a member of staff calls appropriate external housing organisation to begin solving problem within 7 days of expected discharge |
|  | 571 | Every patient's safeguarding status must be captured in the patient record at admission |
|  | 572 | A nurse must capture every patients safeguarding status in the patient record at admission |
|  | 576 | Every patient's safeguarding status must be received from the referring/admitting professional upon admission (social worker, liaison, CMHT, crisis) |
|  | 578 | If a potential safeguarding issue is identified at admission a member of staff must call appropriate external organisation to begin solving problem within 24 hours of admission |
|  | 579 | If a potential safe guarding problem is identified at admission a member of staff must call appropriate external organisation to begin solving problem within 48 hours of admission |
|  | 588 | Every patient's financial responsibilities/difficulties (that may affect discharge) must be captured in the patient record at admission |
|  | 594 | Every patient’s financial responsibilities/difficulties (that may affect discharge) must be received from the patient themselves upon admission (if not already in patient notes) |
|  | 595 | If a potential financial responsibilities/difficulties (that may affect discharge) is identified at admission a member of staff calls appropriate external organisation to begin solving problem within 24 hours of admission |
|  | 596 | If a potential financial responsibilities/difficulties (that may affect discharge) are identified at admission a member of staff calls appropriate external organisation to begin solving problem within 48 hours of admission |
|  | 605 | Ward staff must ensure that benefits are correct/in place before discharge |
|  | 606 | Ward staff must ensure they are aware of the patients benefits status upon admission |
|  | 608 | A nurse must capture every patients physical healthcare needs in the patient record at admission |
|  | 610 | A doctor must capture every patients physical healthcare needs in the patient record at admission |
|  | 612 | Every patient's physical healthcare needs must be requested from the referring agency upon admission (social worker, CMHT, crisis) |
|  | 613 | Every patient's physical healthcare needs must be requested from the patient themselves upon admission (if not already in patient notes) |
|  | 614 | Every patient's unmet community needs are captured in the patient record at admission |
|  | 615 | A nurse must capture every patients unmet community needs in the patient record at admission |
|  | 619 | Every patient's unmet community needs must be requested (if known) from the referring/admitting agency upon admission (social worker, CMHT, crisis) |
|  | 620 | Every patient's is asked about any unmet needs in the community within 24 hours of admission |
|  | 621 | Every patient's is asked about any unmet needs in the community within 48 hours of admission |
|  | 627 | A nurse must capture every patients caregiving responsibilities in the patient record at admission |
|  | 631 | Every patient's caregiving responsibilities must be requested from the admitting/referring professional upon admission (social worker, CMHT, crisis) |
|  | 632 | Every patient's caregiving responsibilities must be requested from the patient themselves upon admission (if not already in patient notes) |
|  | 633 | Details of the patients informal carers must be captured at admission (name, contact, relationship) |
|  | 635 | Details of carers availability must be captured at admission |
|  | 641 | Every patient's involvement with other services must be requested from the referring agency upon admission (social worker, CMHT, crisis) |
|  | 642 | Every patient's involvement with other services must be requested from the patient themselves upon admission (if not already in patient notes) |
|  | 643 | A specific member of staff must be responsible for contacting external organisations that are involved with the patient to let them know about expected discharge and discuss any predicted problems that may affect discharge |
|  | 644 | Nurses must be responsible for contacting external organisations that are involved with the patient to let them know about expected discharge and discuss any predicted problems that may affect discharge |
| Appropriate (median ≥7, DI <1) but with uncertain feasibility (median ≤6.5 ≥3.5, DI <1) |  |  |
|  | 561 | If a potential accommodation problem is identified at admission a member of staff calls appropriate external housing organisation to begin solving problem within 24 hours of admission |
|  | 562 | If a potential accommodation problem is identified at admission a member of staff calls appropriate external housing organisation to begin solving problem within 48 hours of admission |
|  | k11 | K11. Collection of social and financial information for each patient (via external agencies/professionals/the patient and carer) |
| Appropriate (median ≥7, DI <1) but infeasible (median ≤3, DI <1) |  |  |
|  | N/A | No statements ranked in this category |
| Uncertain appropriateness (median ≤6.5, ≥3.5, DI <1) but feasible |  |  |
|  | 553 | A nurse must capture every patients accommodation status in the patient record at admission |
|  | 563 | If a potential accommodation problem is identified at admission a member of staff calls appropriate external housing organisation to begin solving problem within 7 days of admission |
|  | 580 | If a potential safeguarding problem is identified at admission a member of staff must call appropriate external organisation to begin solving problem within 7 days of admission |
|  | 584 | Nurses are responsible for contacting external organisations in relation to safeguarding |
|  | 586 | Doctors are responsible for contacting external organisations in relation to safeguarding |
|  | 597 | If potential financial responsibilities/difficulties (that may affect discharge) problem is identified at admission a member of staff calls appropriate external organisation to begin solving problem within 7 days of admission |
|  | 600 | If potential financial responsibilities/difficulties (that may affect discharge) problem is identified at admission a member of staff calls appropriate external organisation to begin solving problem within 7 days of expected discharge |
|  | 601 | Nurses are responsible for contacting external organisations in relation to financial responsibilities/difficulties (that may affect discharge) |
|  | 622 | Every patient's is asked about any unmet needs in the community within 7 days of admission |
|  | 623 | Every patient's is asked about any unmet needs in the community within 7 days of expected discharge date |
|  | 624 | Every patient's is asked about any unmet needs in the community within 48 hours of expected discharge date |
|  | 637 | A nurse must capture every patients involvement with other services in the patient record at admission |
| Uncertain appropriateness (median ≤6.5, ≥3.5, DI <1) with uncertain feasibility (median ≤6.5, ≥3.5, DI <1) |  |  |
|  | 554 | An administrator must capture every patients accommodation status in the patient record at admission |
|  | 555 | A doctor must capture every patients accommodation status in the patient record at admission |
|  | 556 | A nursing assistant/HCA captures every patients accommodation status in the patient record at admission |
|  | 557 | A patients accommodation information must be captured at clerking in meeting at the latest |
|  | 565 | 1If a potential accommodation problem is identified at admission a member of staff calls appropriate external housing organisation to begin solving problem within 48 hours of expected discharge |
|  | 566 | If a potential accommodation problem is identified at admission a member of staff calls appropriate external housing organisation to begin solving problem within 24 hours of expected discharge |
|  | 567 | Nurses are responsible for contacting external housing organisation |
|  | 568 | Administrators are responsible for contacting external housing organisation |
|  | 569 | Doctors are responsible for contacting external housing organisation |
|  | 570 | Nursing Assistants/HCAs are responsible for contacting external housing organisation |
|  | 573 | An administrator must capture every patients safe guarding status in the patient record at admission |
|  | 574 | A doctor must capture every patients safeguarding status in the patient record at admission |
|  | 575 | A nursing assistant/HCA must capture every patients safeguarding status in the patient record at admission |
|  | 577 | Every patient's safeguarding status must be received from the patient themselves upon admission (if not already in patient notes) |
|  | 581 | If a potential safeguarding problem is identified at admission a member of staff must call appropriate external organisation to begin solving problem within 7 days of expected discharge |
|  | 582 | If a potential safeguarding problem is identified at admission a member of staff must call appropriate external organisation to begin solving problem within 24 hours of expected discharge |
|  | 583 | If a potential safeguarding problem is identified at admission a member of staff must call appropriate external organisation to begin solving problem within 48 hours of expected discharge |
|  | 585 | Administrators are responsible for contacting external organisations in relation to safeguarding |
|  | 587 | Nursing assistants/HCAs are responsible for contacting external organisations in relation to safeguarding |
|  | 589 | A nurse must capture every patients financial responsibilities/difficulties (that may affect discharge) in the patient record at admission |
|  | 590 | An administrator must capture every patients financial responsibilities/difficulties (that may affect discharge) in the patient record at admission |
|  | 591 | A doctor must capture every patients financial responsibilities/difficulties (that may affect discharge) in the patient record at admission |
|  | 592 | A nursing assistant/HCA must capture every patients financial responsibilities/difficulties (that may affect discharge) in the patient record at admission |
|  | 593 | Every patient's financial responsibilities/difficulties (that may affect discharge) must be requested from the referring/admitting professional upon admission (social worker, liaison, CMHT, crisis) |
|  | 598 | If a potential financial responsibilities/difficulties (that may affect discharge) is identified at admission a member of staff calls appropriate external organisation to begin solving problem within 24 hours of expected discharge |
|  | 599 | If a potential financial responsibilities/difficulties (that may affect discharge) are identified at admission a member of staff calls appropriate external organisation to begin solving problem within 48 hours of expected discharge |
|  | 602 | Administrators are responsible for contacting external organisations in relation to financial responsibilities/difficulties (that may affect discharge) |
|  | 603 | Doctors are responsible for contacting external organisations in relation to financial responsibilities/difficulties (that may affect discharge) |
|  | 604 | Nursing assistants/HCAs are responsible for contacting external organisations in relation to financial responsibilities/difficulties (that may affect discharge) |
|  | 611 | A nursing assistant/HCA must capture every patients physical healthcare needs in the patient record at admission |
|  | 616 | An administrator must capture every patients unmet community needs in the patient record at admission |
|  | 617 | A doctor must capture every patients unmet community needs in the patient record at admission |
|  | 618 | A nursing assistant/HCA must capture every patients unmet community needs in the patient record at admission |
|  | 628 | An administrator must captures every patients caregiving responsibilities in the patient record at admission |
|  | 629 | A doctor must capture every patients caregiving responsibilities in the patient record at admission |
|  | 630 | A nursing assistant/HCA must capture every patients caregiving responsibilities in the patient record at admission |
|  | 638 | An administrator must captures every patients involvement with other services in the patient record at admission |
|  | 639 | A doctor must capture every patients involvement with other services in the patient record at admission |
|  | 640 | A nursing assistant/HCA must capture every patients involvement with other services in the patient record at admission |
|  | 645 | Administrators must be responsible for contacting external organisations that are involved with the patient to let them know about expected discharge and discuss any predicted problems that may affect discharge |
|  | 646 | Doctors must be responsible for contacting external organisations that are involved with the patient to let them know about expected discharge and discuss any predicted problems that may affect discharge |
|  | 647 | Nursing assistants/HCAs must be responsible for contacting external organisations that are involved with the patient to let them know about expected discharge and discuss any predicted problems that may affect discharge |
| Inappropriate (median ≤3, DI <1) and infeasible (median <3, DI <1). |  |  |
|  | N/A | No statements ranked in this category |
