# Supplementary material for: Developing Best Practice Guidance for Discharge Planning Using the RAND/UCLA Appropriateness Method
Source: Front Psychiatry. 2021 Dec 3;12:789418. doi: 10.3389/fpsyt.2021.789418 (PMC8680088; doi:10.3389/fpsyt.2021.789418)
Supplement: Supplementary file 2 [file Table_11.docx]

**Supplementary Table 11: Panelists Information (Created by Authors)**

| Panelist name | Job Title | Organisation | Gender | Location | Completed all rounds |
| --- | --- | --- | --- | --- | --- |
| Alison Christie | Ward Manager (Rmn) | NHS Hospital | F | Cornwall | Yes |
| Alison Cobb | Policy And Campaigns Team | Mind | F | London | Yes |
| Kyri Gregoriou | Assistant Director Of Clinical Professional Practice | NHS Hospital | M | Derbyshire | Yes |
| Andrew Grundy | Service User Researcher | Univeristy of Nottingham | M | Nottingham | Yes |
| Umar Kankiya | Mental Health And Mental Capacity Lawyer | Kank Speaks Legal Ltd | M | Grays | Yes |
| Dr Rhiannon Newman | Consultant Psychiatrist | NHS Hospital | F | Cambridgeshire | Yes |
| Peter Pratt | Specialist Pharmacist Advisor To The Mental Health Team | NHS England | M | Sheffield | Yes |
| Dr Abdi Sanati | Consultant Psychiatrist | NHS Hospital | M | East London | No could not make final round |
| Dr Chris Taylor | Clinical Psychologist | NHS Hospital | M | Bury | Yes |
| Dr Nicola Wright | Associate Professor Of Mental Health | University of Nottingham | F | Nottingham | Yes |
